# Genome-Wide Analysis of Nascent Transcription in *Saccharomyces cerevisiae*

**DOI:** 10.1534/g3.111.000810

**Published:** 2011-12-01

**Authors:** Anastasia McKinlay, Carlos L. Araya, Stanley Fields

**Affiliations:** * Department of Genome Sciences; ‡ Department of Medicine; † Howard Hughes Medical Institute, University of Washington, Seattle, Washington 98195

**Keywords:** yeast, *Saccharomyces cerevisiae*, transcription, nuclear run-on assay, high-throughput sequencing, RNA stability

## Abstract

The assessment of transcriptional regulation requires a genome-wide survey of active RNA polymerases. Thus, we combined the nuclear run-on assay, which labels and captures nascent transcripts, with high-throughput DNA sequencing to examine transcriptional activity in exponentially growing *Saccharomyces cerevisiae*. Sequence read data from these nuclear run-on libraries revealed that transcriptional regulation in yeast occurs not only at the level of RNA polymerase recruitment to promoters but also at postrecruitment steps. Nascent synthesis signals are strongly enriched at TSS throughout the yeast genome, particularly at histone loci. Nascent transcripts reveal antisense transcription for more than 300 genes, with the read data providing support for the activity of distinct promoters driving transcription in opposite directions rather than bidirectional transcription from single promoters. By monitoring total RNA in parallel, we found that transcriptional activity accounts for 80% of the variance in transcript abundance. We computed RNA stabilities from nascent and steady-state transcripts for each gene and found that the most stable and unstable transcripts encode proteins whose functional roles are consistent with these stabilities. We also surveyed transcriptional activity after heat shock and found that most, but not all, heat shock-inducible genes increase their abundance by increasing their RNA synthesis. In summary, this study provides a genome-wide view of RNA polymerase activity in yeast, identifies regulatory steps in the synthesis of transcripts, and analyzes transcript stabilities.

Changes in mRNA steady-state levels reflect the combined effect of changes in RNA synthesis and degradation. Thus, measurement of only steady-state transcript levels provides an incomplete view of transcriptional regulation. Proper control of transcription is achieved by regulation at several steps: (1) preinitiation complex assembly; (2) promoter escape (or early elongation); (3) productive elongation; and (4) transcription termination (Fuda *et al.* 2009). Although the spatial distribution of RNA polymerase II can be used to assess transcriptional regulation, polymerase occupancy alone, as detected by chromatin immunoprecipitation, cannot discriminate between polymerases that are simply bound to promoters and those that are transcriptionally engaged. For example, yeast RNA polymerase II associates with many transcriptionally inactive promoters (Radonjic *et al.* 2005).

The nuclear run-on (NRO) assay traditionally has been used to assess the transcription of individual genes (Brown *et al.* 1984; Gariglio *et al.* 1981) and remains the most reliable method of directly measuring transcriptional activity. This method consists of (1) isolation of cell nuclei; (2) isotopic labeling of nascent RNA; and (3) hybridization of labeled RNA to a slot blot, which is then exposed to film. The NRO assay has been scaled up to analyze transcription on a genome-wide basis. Combining this assay with hybridization to nylon macroarrays, García-Martínez *et al.* (2004) and Pelechano *et al.* (2009) detected distinct classes of yeast genes that change their transcription in response to stimuli. By using human cell lines, Core *et al.* (2008) coupled the NRO assay to high-throughput sequencing to provide a genome-wide view of transcriptional activity and identified classes of genes with paused RNA polymerase II near their transcription start sites (TSS).

In this study, we coupled the NRO assay with high-throughput sequencing to analyze transcriptional control in *S. cerevisiae* and found that polymerase recruitment is the predominant form of regulation. However, we demonstrate that, as in metazoans, postrecruitment steps are important for transcriptional regulation in yeast. We used the parallel measurement of nascent transcription and of steady-state RNA to examine the relationship between transcript synthesis, abundance, and stability. We also used this technology to analyze the transcriptional response to heat shock. In summary, we present a high-resolution view of yeast transcription that discriminates among different modes of transcriptional regulation.

## Materials and Methods

### Yeast strains and media

*S. cerevisiae* strain BY4742 was used (Brachmann *et al.* 1998). BY4742 was grown in yeast extract-peptone dextrose medium (2% glucose, 2% peptone, 1% yeast extract) at 30° to exponential growth phase (OD_600_ = 0.5−0.6).

### NRO RNA library construction

NRO assays were performed as described (Garcia-Martinez *et al.* 2004) by the use of approximately 10^7^ cells. *In vitro* transcription was performed with 12 µL of biotin-16-UTP (10 mM; Roche) instead of 13 µL of [^α-33^P]UTP. Total RNA was extracted with hot phenol, treated with Turbo DNase I that was passed through CentriSpin columns (Princeton Separations, Adelphia, NJ). An aliquot of total RNA was used for total RNA library construction (see *Materials and Methods*, Total RNA library construction). Biotinylated RNA was precipitated from total RNA with Dynabeads MyOne Streptavidin C1 (Invitrogen, Carlsbad, CA). Binding was performed in 5 mM Tris-HCl (pH 7.5), 0.5 mM ethylene diamine tetra-acetic acid and 1 M NaCl for 20 min at 42° and 2 hr at room temperature with rotation as described (Patrone *et al.* 2000). Beads were first washed three times with 800 µL of binding buffer and then washed twice with 800 µL 2x saline sodium citrate (SSC)/15% formamide buffer for 15 min, followed by a 5-min wash in 800 µL 2x SSC. The beads were then washed twice more with 2x SSC with 15% formamide followed by a wash with 2x SSC and a final wash with TE buffer. RNA was eluted from beads by adding 50 µL of 10 μM ethylene diamine tetra-acetic acid /95% formamide and incubation at 65° for 5 min. Eluted RNA was precipitated, fragmented (Ambion Fragmentation kit; Ambion, Austin, TX), and used for strand-specific Illumina sequencing library preparation as described (Parkhomchuk *et al.* 2009). In brief, this protocol relies on marking the second strand with dUTP during synthesis followed by its degradation after adaptor ligation and size selection. A comparative analysis of strand-specific RNA sequencing methods (Levin *et al.* 2010) identified this method as leading by performance across tested criteria. DNA libraries were submitted for 36-bp single-end sequencing.

#### For 5′-end sense strand sequencing

PE adapters sequences:

5′ P-GATCGGAAGAGCGGTTCAGCAGGAATGCCGAG
5′ ACACTCTTTCCCTACACGACGCTCTTCCGATC*T

PE PCR primers:

5′ AATGATACGGCGACCACCGAGATCTACACTCGGCATTCCTGCTGAACCGCTCTTCCGATC*T
5′ CAAGCAGAAGACGGCATACGAGATCGGTCTCTTTCCCTACACGACGCTCTTCCGATC*T

PE sequencing primer:

5′ TACACTCGGCATTCCTGCTGAACCGCTCTTCCGATCT

#### For 3′-end sense strand sequencing.

PE adapters sequences:

5′ P-GATCGGAAGAGCGGTTCAGCAGGAATGCCGAG
5′ ACACTCTTTCCCTACACGACGCTCTTCCGATC*T

PE PCR primers:

5′   AATGATACGGCGACCACCGAGATCTACACTCTTTCCCTACACGACGCTCTTCCGATC*T
5′   CAAGCAGAAGACGGCATACGAGATCGGTCTCGGCATTCCTGCTGAACCGCTCTTCCGATC*T

PE sequencing primer:

5′ ACACTCTTTCCCTACACGACGCTCTTCCGATCT

* indicates a phosphorothioate bond.

### Total RNA library construction

Total RNA was treated with Turbo DNase I, fragmented (Ambion Fragmentation kit) and used for Illumina sequencing library preparation as described (Parkhomchuk *et al.* 2009). In brief, approximately 50 ng of total RNA was used for first-strand synthesis with random hexamers. After subsequent removal of dNTPs with NucAway Spin Columns (Ambion), second-strand synthesis was performed in the presence of dUTP. Double-stranded cDNA was purified with the PureLink PCR purification kit (Invitrogen) and subjected to end repair, A-tailing, and adaptor ligation steps. DNA libraries were fractionated on a nondenaturing 1x TBE, 6% acrylamide gel (Invitrogen), and fragments between 250 and 350 bp were excised from the gel and eluted by incubation in TE at room temperature with vigorous shaking for 30 min. Uridine-containing, second-strand synthesis products were digested with Uracil-N-Glycosylase (Applied Biosystems). Libraries were amplified via 16 PCR cycles. PCR products were cleaned (Invitrogen PureLink, Agencourt AMPure XP; Beckman Coulter Genomics, Danvers, MA) and submitted for 36-bp single-end sequencing.

### Assessment of the NRO assay performance for the enrichment of nascent RNA

Two different regions of *Arabidopsis thaliana* ELF3 gene were transcribed *in vitro* either in the presence of UTP or B16UTP. B16UTP- and UTP-labeled transcripts from two regions were mixed at approximately 10^9^ copies and added to 50 μg of yeast total RNA as spike-in controls. The selection experiments were performed in triplicates to obtain average Cp values. Fold of enrichment (*f*) of B16UTP-RNA over nonspecifically bound UTP-RNA on streptavidin beads was calculated on the basis of the differences in their Cp values and adjusted by the differences in Cp values observed in their corresponding inputs:

$$f=\frac{2^{NRO(UTP-RNA).Cp-NRO(B16UTP-RNA).Cp}}{2^{Input(UTP-RNA).Cp-Input(B16UTP-RNA).Cp}}$$

### Sequence alignment

We aligned quality control-filtered reads from the NRO and total RNA libraries to the UCSC sacCer2 reference genome by using *Maq* (http://maq.sourceforge.net) with default parameters and filtered to retain high confidence alignments (mapping quality ≥ 30). The quality-filtered alignments were used to compile a strand-specific measure of abundance for transcribed bases in the genome, normalized by the total number of bases acquired per experiment. Alignments from biological replicates were merged by use of the *mapmerge* function in *Maq* and processed equally. All reads were 36 bp long.

### Definition of mappable bases in the genome

To define bases in the genome that can be unequivocally covered by reads, we identified all unique 36 base sequences from both strands of the reference assembly. We defined mappable bases as all positions covered by these sequences.

### Calculation of gene activity

The normalized per-base read depth from NRO and total RNA libraries was mapped to a collection of previously annotated features that do not overlap in the same strand, including genes with known transcription start and end sites, as well as genes with unknown transcription starts and ends, and noncoding RNAs (Nagalakshmi *et al.* 2008). For each feature, transcriptional activity was measured as the average normalized read depth in mappable, nonintronic bases (“read density”). Similarly, we derived a background RNA sequencing rate for each data set as the average minimum read depth in a collection of intergenic regions. The significance of the read density for each feature was calculated against the Poisson distribution of read depth per base expected from the background. A *P*-value significance cutoff of 0.01 was applied.

### Transcriptional AUC analysis

The cumulative read depth along each transcript was calculated to examine the rate of transcription along transcript models. For each position *i* from the TSS, the cumulative read depth *Ci* represents the sum of bases sequenced from the TSS to *i*. The cumulative read depth at each position (*Ci*) was normalized by the total read bases in the transcript model to obtain the fraction (*Fci*) of the total read depth in the model acquired up to each position *i*. Transcript model sizes were homogenized to allow comparisons between models of different length. Sizes were adjusted by maintaining the per-base values from the first and last 100 bp of the model and adjusting the intervening values to a 1000-bp segment. Thus, the fractional cumulative NRO read depths along the uniformly sized models represent the amount of transcriptional activity that has occurred as a function of position in the 5′ to 3′ direction of the transcript model.

For each gene, we calculated the area under the curve (AUC) from uniformly sized models by using normalized (*Fci*) and non-normalized (*Ci*) cumulative read depth data and 10,000 simulated AUCs (non-normalized). Simulations were performed by randomly placing the observed number of transcript reads within the model and summing the contributions in cumulative read depth of each alignment to the size-adjusted transcript model. The significance of the observed AUC for each transcript model was assessed against the distribution of simulated AUCs under the null hypothesis that transcription occurs evenly throughout the model. Genes were ranked by their normalized AUC values (*i.e.* based on *Fci* curves).

### Gene Ontology analysis

Gene ontology analysis for subsets of gene lists was performed with the GO TermFinder (http://yeastgenome.org/) with a *P*-value significance cutoff of 0.01 and a 5% FDR threshold. Analysis of GO term enrichment near the top and bottom of genes ranked by their ratios of transcription activity and transcript abundance (supporting information, Table S3 and Table S4) were performed with GOrilla (Eden *et al.* 2009).

### Data availability

Raw Illumina reads are available at http://www.ncbi.nlm.nih.gov/geo as series GSE33136.

## Results

### Genome-wide assessment of transcriptional activity and transcript abundance

**Figure 1:**
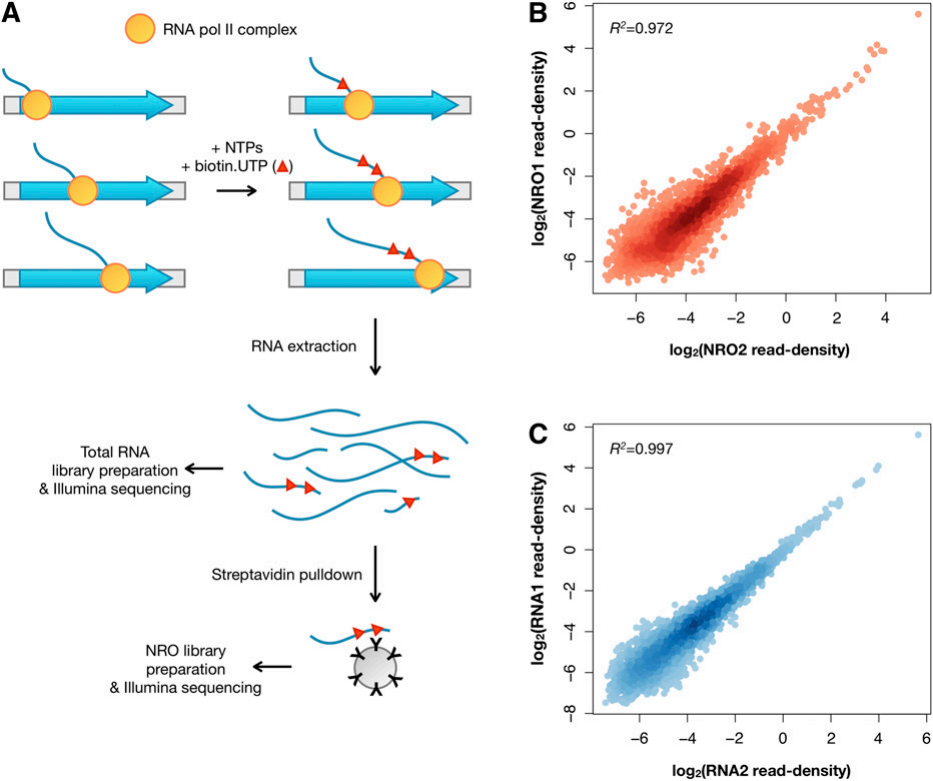
A strategy for the genome-wide analysis of nascent transcription. (A) Schematic of the NRO assay. Transcription complexes are stalled on genomic DNA by depleting endogenous nucleotide triphosphates via permeabilization of yeast cells with sarcosyl. Reactivation of transcription is achieved by providing biotin-UTP and other nucleotides and switching transcription reactions from ice to 30° for 5 min, followed by extraction of total RNA that contains both steady-state and nascent biotinylated transcripts. A fraction of the total RNA is used for total RNA library preparation. Newly synthesized biotinylated RNAs are isolated from total RNA via streptavidin pull-downs to prepare the NRO sample. Both NRO and total RNA strand-specific Illumina sequencing libraries are constructed and submitted for 36 bp single-end sequencing. (B, C) After mapping reads to the reference genome and quality-filtering alignments, the read density is computed from NRO and total RNA data in 4,285 genes with fully distinguishable transcript models (*i.e.* that do not overlap on the same strand). These measurements of transcript synthesis and transcript abundance for each gene are found to be robust between NRO (B) and total RNA (C) replicates.

We established a genome-wide NRO assay for yeast consisting of the following steps: (1) permeabilization of yeast cells with sarcosyl to deplete endogenous nucleotides and stall transcription complexes on chromosomes; (2) reactivation of transcription to extend and label newly synthesized RNA transcripts with biotin-modified UTP; (3) isolation of biotin-modified RNA on streptavidin beads; (4) elution of enriched RNA from beads; and (5) construction of strand-specific high-throughput sequencing libraries (Figure 1A). To assess the extent of nonspecific binding of RNA to streptavidin beads and to optimize conditions for the NRO steps, we performed parallel NRO reactions with biotin-UTP and, as a control, with UTP. Triplicates of both reactions were processed in parallel through the steps of binding, washing, and eluting from streptavidin beads. To measure the enrichment of biotinylated RNA, we performed quantitative PCR on the eluted RNA as well as on the input RNA. The signals from biotin-UTP-containing RNA and UTP-containing RNA were first normalized to their corresponding signal from input (see the Materials and Methods) and then used to calculate the fold enrichment of biotinylated transcripts over nonbiotinylated ones for three highly expressed yeast genes (*RDN18*, *ACT1*, and *RPL28*). The NRO assay conditions were optimized to reduce total RNA background to 4%–11% (Table S1). In addition, we performed NRO reactions spiking in controls consisting of two regions of the *Arabidopsis thaliana* ELF3 RNA that had been transcribed *in vitro* either in the presence of UTP or biotin-UTP (Table S2). Together these quantitative PCR experiments demonstrated that total RNA represents between 2.6% and 11% of the NRO libraries and that therefore nascent RNA constitutes between approximately 89% and 97% of the NRO samples. By using a standard curve from known amounts of *in vitro* synthesized, biotin-labeled RNA, we estimate that the starting amount of biotinylated nascent transcripts in RNA extracts before affinity selection comprises approximately 0.05%. Thus, we calculate that after selection nascent RNA has been enriched by a factor of at least 89/0.05, or approximately 1,800-fold, relative to its starting level.

To examine RNA polymerase II activity and monitor transcript abundance throughout the genome, we constructed both NRO and total RNA libraries. Total RNA libraries were prepared from aliquots of the biotin-UTP-treated NRO sample by excluding the steps of nascent RNA selection on streptavidin beads (Figure 1A). Strand-specific Illumina sequencing libraries were built from RNA isolated from three independent cultures of log phase yeast (Parkhomchuk *et al.* 2009). We acquired 63 million and 83 million 36 base-pair reads from the NRO and total RNA libraries, respectively. After aligning reads to the genome, we filtered out low-quality alignments and rRNA sequences; because we carried out the protocols without an rRNA depletion step, rRNA sequences accounted for more than 90% of the total reads. After filtering, approximately 2.5 million uniquely mapped NRO reads and approximately 1 million total RNA reads were left (Table S3). This combined approach allowed us to measure the amount of nascent transcripts as well as steady-state transcripts in parallel.

To analyze sequencing results for both NRO and total RNA samples, we defined read depth as the number of sequencing reads acquired per base, normalized by the total bases sequenced for that sample. We summed the read depth values for each mappable, non-intronic base within a transcript model and divided this sum by the total number of these bases within that transcript to calculate read density. Both read depth and read density are presented in units of read depth per million bases acquired to obtain higher scale values.

We focused our analysis on a set of 4,285 genes with known TSSs and termination sites (Nagalakshmi *et al.* 2008) and that do not overlap on the same strand. The reproducibility of the transcriptional activity and steady-state levels as determined from read densities in replicates of NRO and total RNA libraries was robust (Pearson’s *r^2^* > 0.97 and *r^2^* > 0.99, respectively; Figure 1, B and C). Therefore, we merged reads from biological replicates to obtain a single NRO data set and a single total RNA data set for subsequent analyses. Of the 4,285 nonoverlapping transcript models, 2,717 and 2,802 genes were transcriptionally active in the NRO and total RNA libraries, respectively, as measured against the background from intergenic regions (*P* < 0.01; see the Materials and Methods). Our measurements of nascent transcription showed moderate correlation with previous genome-wide estimates of transcriptional activity derived from hybridization of radiolabeled nascent transcripts to nylon macroarrays (Spearman’s ρ = 0.53; Figure S1A). This correlation is comparable with that between previous measures of transcriptional activity obtained via the use of NRO approaches (Spearman’s ρ = 0.61; Figure S1B) (Garcia-Martinez *et al.* 2004; Pelechano *et al.* 2009). Excluding the rDNA cluster, we detected no large regions (>3 kb) in the yeast genome with extensive synthesis of nascent RNA (*Z* > 3.1; Figure S2).

Because the NRO assay reflects the activity of RNA polymerases on transcribed genes, we compared the transcription data from the NRO sample to the genome-wide distribution of RNA polymerase II in yeast (Lefrancois *et al.* 2009), and found a low but significant correlation (Pearson’s *r^2^* = 0.41, *P* < 2.2 × 10^−16^; Figure S3A). The modest correlation between polymerase occupancy and transcriptional activity has been previously reported (Rodriguez-Gil *et al.* 2010) and might result from differences in the dynamic range of the protocols (as suggested by Rodriguez-Gil *et al.* 2010) or the inability of the NRO assay to precisely assign the positions of transcribing polymerases, because fragmentation of RNA was performed after its affinity selection. As expected, total RNA also correlated with polymerase occupancy, but to a lesser extent (Pearson’s *r^2^* = 0.37, *P* < 2.2 × 10^−16^; Figure S3B). This analysis corroborates that RNA polymerase occupancy is a better predictor of transcriptional activity than of transcript abundance.

Although pre-mRNA splicing occurs co-transcriptionally in yeast, we observed greater intronic read depths in the NRO libraries than in the total RNA libraries (Figure S4A), and this increase was not caused by increased transcript levels. Moreover, splicing was much less abundant in the NRO libraries than in the total RNA libraries (Figure S4B), further arguing that the NRO libraries are enriched for nascent transcripts.

### Nascent transcription is enriched near TSS

**Figure 2:**
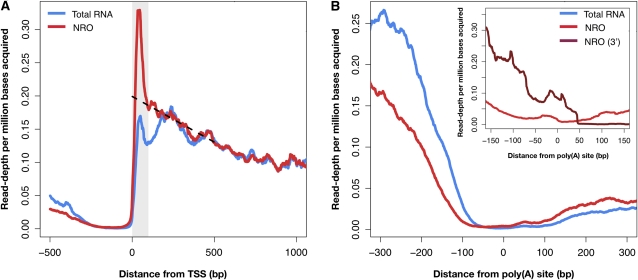
Distribution of RNA polymerase activity relative to TSSs and termination sites. (A) The average sense read depth in the NRO (red) and total RNA (blue) libraries along 2,530 genes that are transcriptionally active in both libraries is plotted as a function of distance from TSSs. NRO libraries display a read depth peak within 100 bp 3′ from the TSS (highlighted in gray) with a peak maximum at approximately 50 bp downstream of their TSSs. Dashed line extends the slope of sequencing reads obtained along the transcript length (between 100-500 bp) toward the TSS to extrapolate read depth signal at the 5′ ends of transcripts. (B) Distribution of RNA polymerase activity relative to poly(A) sites in the 5′-end and 3′-end sequenced NRO libraries. The average sense read depth in the NRO (red) and total RNA (blue) libraries in transcriptionally active genes is plotted as a function of distance from poly(A) cleavage sites. A higher read depth near transcript termination sites is found for the total RNA libraries as compared to the NRO libraries. Inset: An additional strand-specific NRO library was sequenced from the 3′ end to compare sense strand read depth information to that from 5′ end libraries. Sense read depth from 5′ end (light red) and 3′ end (dark red) nascent RNA libraries is plotted as a function of distance from poly(A) sites for convergently transcribed genes (*N* = 2000).

Analysis of RNA polymerase II occupancy data from high-density tiling microarrays demonstrated that only highly expressed yeast genes (~16% of all genes) have an even distribution of RNA polymerase II throughout their coding region, and that a comparable fraction (~18%) have an enrichment of the polymerase near their 5′ ends (Venters and Pugh 2009). This result and recent analysis of polymerase-associated RNAs suggests that transcription in yeast, as in metazoans, can be subject to rate-limiting regulatory steps in addition to polymerase recruitment and activation (Churchman and Weissman 2011). To analyze RNA polymerase II activity within genes, we examined read depth along a set of 2,530 transcript models of genes that were transcriptionally active in both the NRO and total RNA libraries. In contrast to the total RNA sample, the NRO sample showed an enrichment of reads near the 5′ ends of transcript models, with a read depth peak occurring approximately 50 bp downstream of the TSS (Figure 2A), as has been observed in human and *Drosophila* cells (Core *et al.* 2008; Rasmussen and Lis 1993). Sarkosyl treatment in the NRO assay allows RNA polymerases that are either transcribing or paused to incorporate nucleotides upon transcription reactivation (Rougvie and Lis 1990) but prevents further rounds of initiation (Hawley and Roeder 1985). Thus, the peak in read depth near TSSs (Figure 2A) likely indicates an accumulation of paused RNA polymerases at 5′ ends. Because we carried out the enrichment of nascent RNA before its chemical fragmentation, all nascent transcripts detected in the NRO assay should have originated at their TSSs. This feature of the protocol results in nested sets of transcripts initiating at their 5′ end, with progressively fewer transcripts in each set containing bases further toward the 3′ end, even for cases with relatively equal distribution of RNA polymerases along the gene. Indeed, we observe a gradual decrease in read depth beginning approximately 100 bp downstream from the TSS (Figure 2A; dashed line). The slope projects a read depth peak toward the TSS of 0.20 resulting from the 5′ end being present in all the nascent transcripts. That the promoter proximal peak rises sharply near the TSS to a height more than 1.5-fold greater than the extrapolated value provides evidence for an enrichment of nascent transcripts, and hence RNA polymerase activity, near TSSs under NRO conditions. To assure that the observed promoter-proximal peak is a feature of yeast transcription, we performed additional analyses and ruled out the effect of nonspecific RNA binding (Figure S5), increased average read depth of shorter transcripts (Figure S6A), and sequencing biases (Figure S6B) as possible contributors to the increased read depth near 5′ ends of yeast transcripts.

Near transcript termination sites, total RNA libraries show greater read depths than NRO libraries (Figure 2B), which is consistent with the fact that most nascent RNAs are not full length (Carrillo Oesterreich *et al.* 2010). Because our protocol acquires sense strand information from the 5′-most 36 bases of fragments, we were not able to detect a peak of nascent transcription near poly(A) sites, as was previously shown for human and yeast gene transcription (Carrillo Oesterreich *et al.* 2010; Core *et al.* 2008). To address this issue, we prepared a new NRO library from which we specifically sequenced the 3′ end bases of transcripts and found a small peak of transcription consistent with poly(A) cleavage (Figure 2B; inset).

### RNA polymerase II activity is consistent with transcriptional control at postrecruitment steps

**Figure 3:**
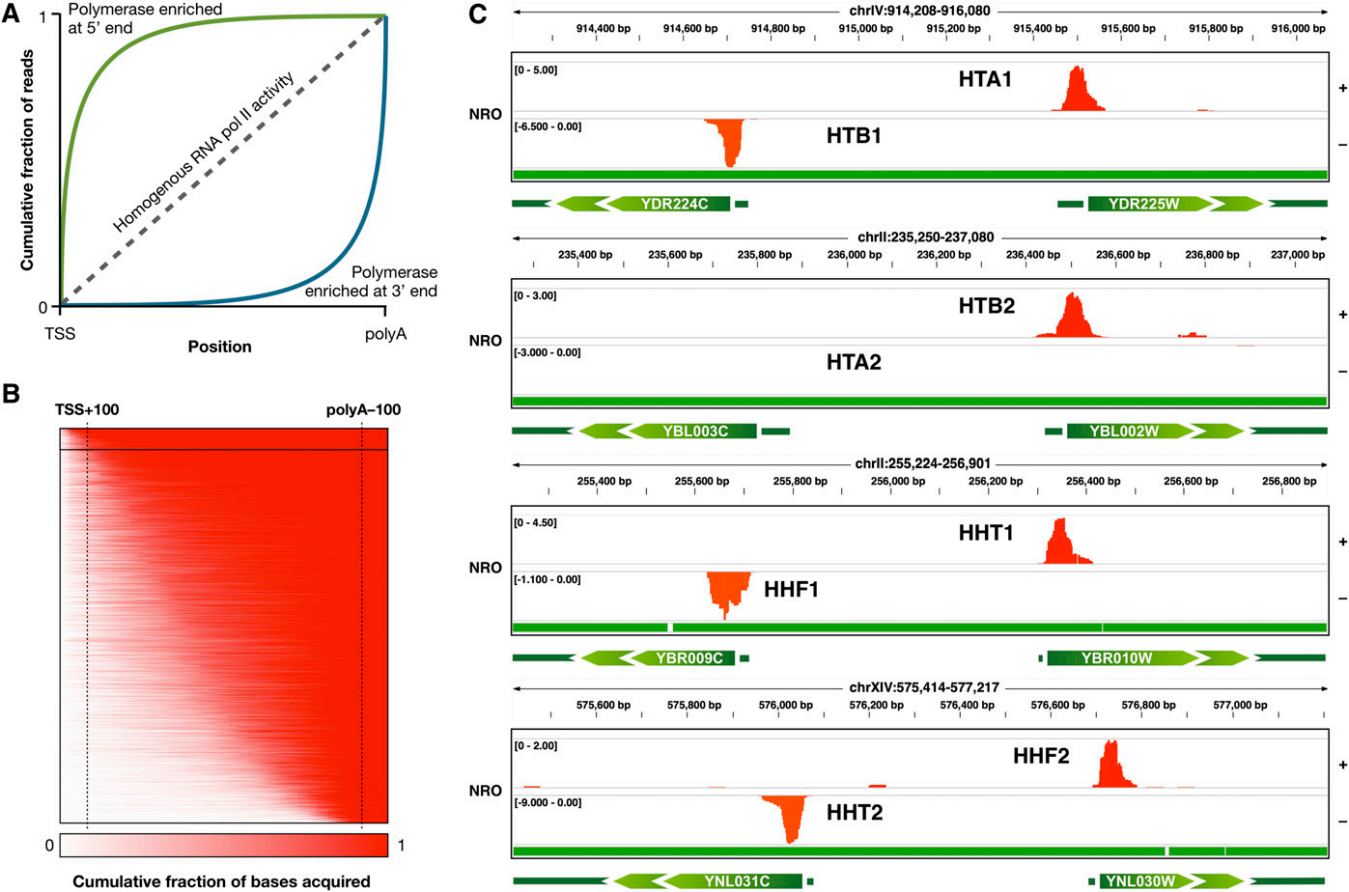
Identification of paused transcripts from nascent RNA libraries. (A) Graph depicts the expected accumulation of reads as a function of distance from TSS for transcripts enriched with polymerase at their 5′ end (green line) or 3′ end (blue line) or transcripts with homogenous RNA polymerase II activity (dashed line). (B) AUC ranking of genes. For each transcript model, the fraction of the total read depth acquired per position along the direction of transcription was computed. This fractional, cumulative read depth within transcript models was adjusted to a uniform model by redistributing values to reflect the read depth in the 5′-most 100 bp, 3′-most 100 bp, and the intervening sequence (marked with vertical dashed lines). Genes were ranked by the AUC of fractional, cumulative read depth in the uniform models. The fraction of the total read depth acquired for each transcript model is shown as a function of distance along the uniform model, where red represents 100%. Genes in the top 5% are highlighted within the black box and contain the histone loci. (C) IGV genome browser views (http://www.broadinstitute.org/igv) of NRO read depth in the four divergently transcribed histone loci. With the exception of *HTA2*, core histones exhibit strong promoter-proximal pausing in NRO libraries. Normalized read depth is shown in the plus and minus strands as positive and negative values, respectively. Read depth range is indicated between brackets. Transcript models are schematized with green arrows indicating coding sequences. Mappable bases are marked green in the track underneath IGV views.

In principle, the capacity to measure transcriptional activity for each base in the genome should allow us to distinguish rate-limiting steps in transcription other than transcription initiation. However, because read depth in high-throughput sequencing of RNA libraries is noisy (Ingolia *et al.* 2009), this goal is difficult. We sought to address this challenge by analyzing how reads accumulate along transcript models. Genes with a relatively homogenous distribution of active polymerases should display a linear increase in the cumulative fraction of reads along a transcript, whereas genes with polymerases predominantly near their 5′ ends or near their 3′ ends generate deflections from this line (Figure 3A). For a gene in which most reads are acquired near the 5′ end of the transcript, the cumulative fraction curve climbs sharply to its maximum value and flattens. Thus, the AUC for the cumulative fraction of reads in genes with polymerase activity concentrated at the 5′ end is larger than for those that are not paused or that accumulate reads at the 3′ end.

To calculate the AUC for each gene, we first calculated the cumulative fraction of reads acquired at each position. We adjusted all transcript models to a uniform length, corresponding to the number of reads obtained for the 5′-most 100 bp, the 3′-most 100 bp, and the intervening sequence. We ranked genes by AUC to identify those with distinct distributions of RNA polymerase II activity along the length of their transcripts, which might correlate with distinct modes of transcriptional regulation (Figure 3B). AUC ranking of transcripts revealed that the top 5% of genes are enriched for nucleosome components (*P* = 7.15 × 10^−5^), chromatin assembly and disassembly genes (*P* = 0.001), helicases (*P* = 0.007), and ATPases (*P* = 0.008). Histone genes drive this enrichment because seven of the eight core histone genes demonstrated strong evidence of accumulation of polymerases at their 5′ ends, which could reflect promoter-proximal pausing (Figure 3C). Given that transcription of histone genes is tightly limited in the cell cycle to early *S* phase, these data suggest that polymerase pausing could play a role in assuring rapid induction of histone synthesis just before the DNA synthesis (Hereford *et al.* 1981), consistent with the previous observation of paused RNA polymerase II on the *Drosophila* histone H3 gene (Li *et al.* 1996). Similarly, a Poisson-based test, as used in Core *et al.* 2008, characterized up to 20% of yeast transcripts as paused, suggesting that their expression may be subject to postinitiation transcriptional control (Figure S7). However, previous chromatin immunoprecipitation analysis of yeast did not provide evidence for widespread polymerase pausing (Venters and Pugh 2009). The evidence of polymerase pausing from the NRO results may partially reflect the protocol used because fragmentation of RNA after affinity selection can make it difficult to precisely assign the position of polymerase.

The AUC ranking allowed further identification of potential rate-limiting steps in transcription. This analysis identified not only genes that acquired the majority of their reads near their TSSs but also genes with low and middle AUC values. Genes with the lowest AUC values exhibited an accumulation of reads near their 3′ ends, which may reflect regulation at the level of transcription termination (Figure S8). The middle range of the AUC-ranked list includes transcripts in which the cumulative fraction of reads increased linearly. In addition, the intermediate range includes transcripts with increased read densities in both the promoter-proximal region and near the 3′ end of the transcript (Figure S8). Such transcripts could be targets of regulation at both promoter escape and transcriptional termination. Finally, transcripts in which nascent transcription is concentrated in the middle of the transcript are also found in this range. Although some of the patterns of read depth could be attributable to stochastic variations in fragment ligation, amplification or sequencing, the observation of transcriptional regulation occurring subsequent to initiation, pausing and release from pausing is consistent with recently observed patterns of RNA polymerase II activity derived from polymerase-associated RNAs (Churchman and Weissman 2011).

### Nascent antisense transcription is not predominantly driven by bidirectional promoters

Because recent genomic analyses have shown substantial antisense transcription in yeast (Nagalakshmi *et al.* 2008; Neil *et al.* 2009; Xu *et al.* 2009; Yassour *et al.* 2010), we asked whether we could detect the presence of antisense transcripts in the NRO and total RNA libraries. We found evidence of antisense transcription in 310 genes in the NRO libraries and in 135 genes in the total RNA libraries (Figure S9). The increased prevalence of antisense transcripts in the NRO sample likely relates to their instability and their consequent loss from the pool of total RNA.

**Figure 4:**
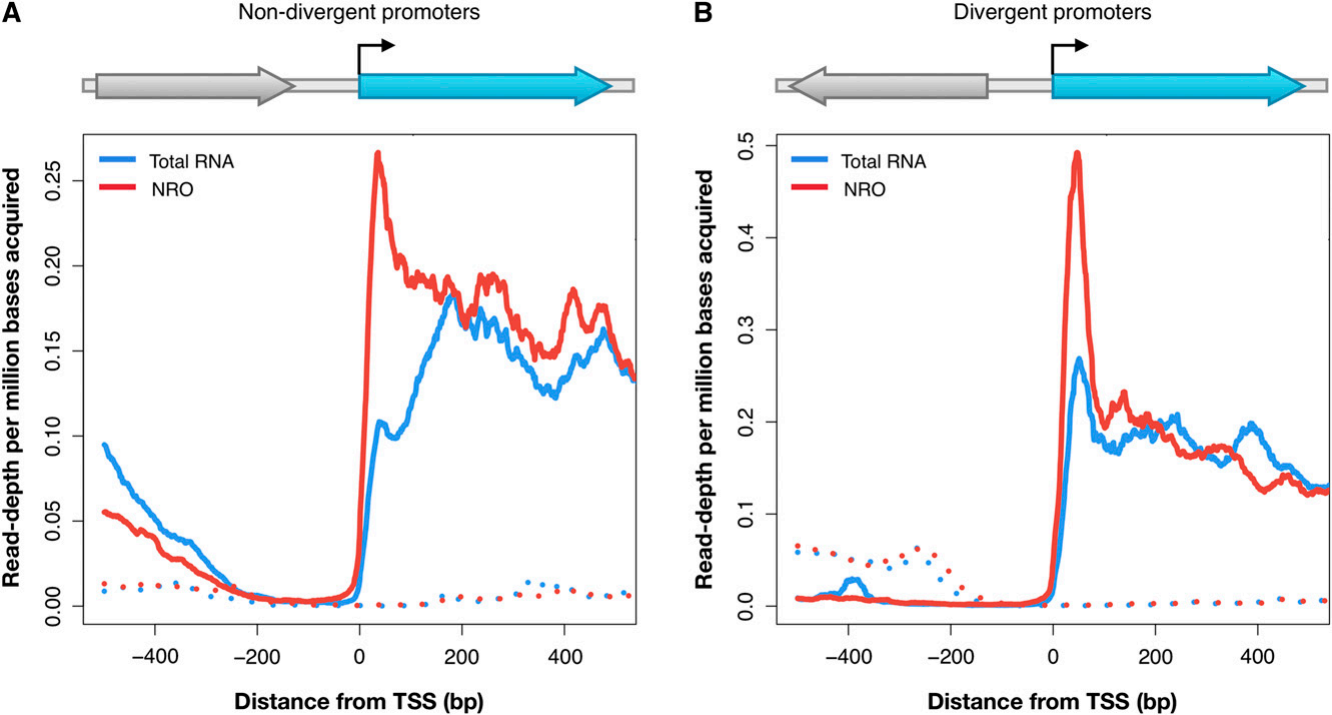
Bidirectional transcription from TSSs is not predominant in yeast. Sense (solid line) and antisense (dotted line) read depths are shown near the TSSs of reference genes (blue) with nondivergent (A) and divergent (B) promoters. Schematics indicate the orientations of transcripts upstream of reference genes with solid lines annotating sense and dotted lines antisense transcription. NRO and total RNA read depths are shown in red and blue, respectively. Read depth is classified as antisense and sense with respect to the 3′ reference gene in blue. In both types of promoters, NRO libraries display predominant sense transcription and a read depth peak approximately 50 bp downstream of their TSSs but do not show significant transcription in the antisense orientation.

We asked whether bidirectional transcription from single promoters, as has been recently reported in yeast (Neil *et al.* 2009; Xu *et al.* 2009), underlies the increased antisense transcription. We classified each promoter with respect to its upstream adjacent gene as either nondivergent (Figure 4A), in which the upstream gene is encoded on the same strand; or divergent (Figure 4B), in which the upstream gene is encoded on the opposite strand. We examined sense and antisense read depth in the 5′ flanking regions of the reference promoters (Figure 4, A and B, in blue) for the two configurations. For nondivergent promoters, very few antisense reads accumulated in the 200-bp 5′ to the TSSs of the reference promoters (Figure 4A), indicating that these promoters drive little antisense transcription. Similarly, for divergent promoters, very few antisense reads accumulated in the 100-bp 5′ to the TSSs (Figure 4B). The increasing antisense read depth beyond 100 bp reflects the activity of distinct promoters for the upstream genes, consistent with separate preinitiation complexes at divergently transcribed genes leading to a detectable signal (Venters and Pugh 2009).

### Relationship between RNA synthesis, abundance, and stability

**Figure 5:**
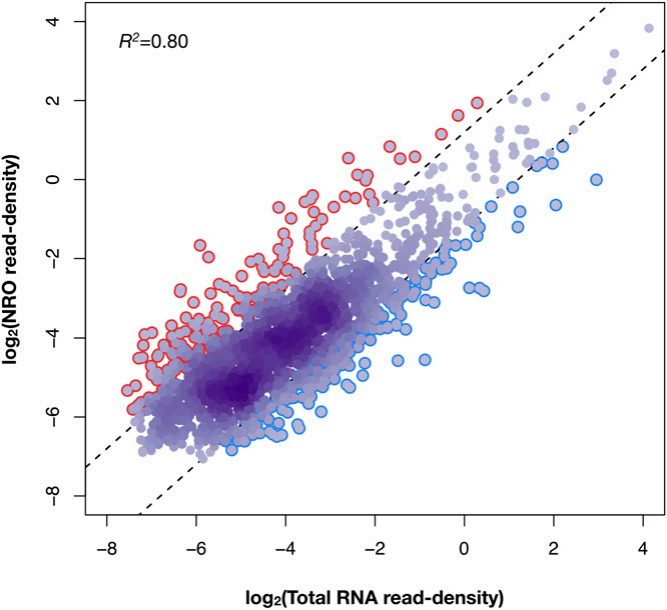
Contribution of transcript synthesis to transcript abundance and inference of transcript stability. Read density in the NRO and total RNA libraries is plotted for a set of 2,414 genes with significant transcription from 201 bp downstream of TSS. Transcript synthesis and transcript abundance, measured as read density within this region of transcripts in NRO and total RNA libraries, respectively, are strongly correlated. To estimate nascent transcript stability, we calculated the ratio of the transcript synthesis to transcript abundance for each gene. Genes in the top and bottom deciles of these ratios are circled in red and blue, respectively.

Having measured both transcriptional activity and steady-state levels of RNA, we sought to use the genome-wide data to explore the relationship between RNA synthesis, abundance, and stability. For this analysis, we chose to estimate transcriptional activity and steady-state RNA by calculating the read density excluding data within 200 bp of the TSS; in this way, we avoided analyzing transcripts that may have prematurely terminated. We compared the NRO-derived transcriptional activity with total RNA-derived steady-state levels and found a significant correlation (Pearson’s *r^2^* = 0.80), suggesting that this measure of transcriptional activity accounts for approximately 80% of the variance in transcript abundance (Figure 5).

To estimate the stability of nascent transcripts, we calculated the ratio of the transcriptional activity to the steady-state level for each gene. High ratios are consistent with short-lived transcripts, because much of their nascent RNA does not reach the steady-state pool. Gene Ontology analysis revealed that genes with the greatest ratios of transcriptional activity to steady state levels (Figure 5, in red) are enriched for transcripts involved in translational elongation (*P* = 7.0 × 10^−6^), drug transport (*P* = 5.1 × 10^−6^), signal transduction (*P* = 1.0 × 10^−4^), and protein amino acid phosphorylation (*P* = 1.5 × 10^−4^; Table S4). Short-lived transcripts are capable of rapid adjustments in abundance to assure quick responses to stimuli (Friedel *et al.* 2009; Grigull *et al.* 2004; Narsai *et al.* 2007; Wang *et al.* 2002). Conversely, genes with the lowest ratios of transcriptional activity to steady state levels (Figure 5, in blue) represent more stable nascent transcripts, and are enriched for structural constituents of the ribosome (*P* = 6.4 × 10^−8^) and genes involved in nucleosome assembly (*P* = 3.6 × 10^−4^; Table S5). Thus, our estimates of the stability of nascent transcripts agree with the expected stabilities of transcripts based on the functions of their encoded proteins, as observed previously (Friedel *et al.* 2009; Grigull *et al.* 2004; Narsai *et al.* 2007; Wang *et al.* 2002).

The absolute half-lives of mRNAs have traditionally been measured over a time course after the inhibition of transcription by chemical or thermal inactivation of RNA polymerase II (Grigull *et al.* 2004; Wang *et al.* 2002). However, mRNA half-lives estimated by different groups by the use of similar types of experiments (Grigull *et al.* 2004; Wang *et al.* 2002) show little correlation (Pearson’s *r^2^* = 0.17; Figure S10A). We observed no correlation between the estimated stabilities of nascent transcripts in our study and previously measured mRNA decay rates (Grigull *et al.* 2004; Wang *et al.* 2002) (Figure S10, B and C). These discrepancies could at least in part be attributable to differences in the stability of nascent transcripts *vs.* that of mRNAs. In addition, the temperature shift to halt transcription might induce changes in RNA folding and thus affect estimates of mRNA half-lives. Finally, technical differences in the methodologies used may be contributing to these differences. For example, microarray measurements in of relative transcript abundance are subject to errors caused by differences in hybridization efficiencies. Similarly, sequencing biases introduce errors into our measurements of both transcriptional activity and steady-state levels of mRNAs and these errors may be compounded in the calculation of ratios.

### Changes in yeast gene transcription in response to heat shock

Several genome-wide studies have identified yeast genes that respond to temperature shock by elevating their transcript levels (Gasch *et al.* 2000; Hahn *et al.* 2004). We sought to examine the extent to which the effect of heat shock on transcript abundance occurs via the up-regulation of transcriptional activity. Thus, we built NRO libraries (“NRO [HS]”) and total RNA libraries (“total RNA [HS]”) from RNA isolated from six independent cultures of log phase yeast under heat shock conditions (20 min at 39°; Table S6).

**Figure 6:**
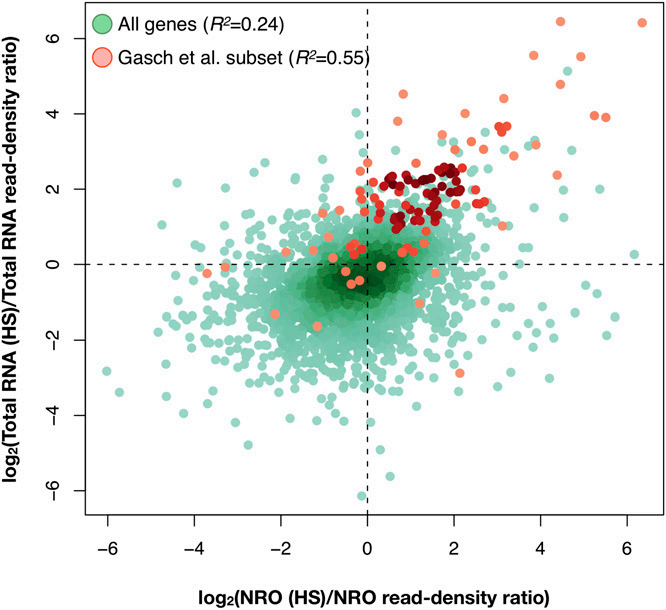
Transcript synthesis and abundance response to heat shock treatment. Ratios of read densities before and after heat shock are plotted for the NRO and total RNA libraries with all significantly expressed yeast genes shown in green and heat shock-responsive genes (≥3x increase in abundance upon heat shock treatment in Gasch *et al.* 2000 data set) shown in red.

To estimate the effect of heat shock on RNA abundance and transcription activity, we calculated the log-transformed ratios of read density values after and prior to heat shock for total RNA (total RNA [HS]/total RNA) and for nascent RNA (NRO [HS]/NRO). For total RNA, log-space ratios greater than zero represent transcripts with increased RNA abundance upon heat shock treatment, whereas those less than zero indicate decreased RNA abundance. For nascent RNA, log-space ratios greater than zero identify genes whose transcription was up-regulated upon heat shock, whereas those less than zero indicate those down-regulated (Figure 6). This analysis reveals that 304 (9%) yeast genes responded to heat stress by increasing RNA abundance by 2-fold or more (Table S7). Approximately 65% of those genes showed an increase in nascent RNA synthesis in response to heat shock, indicating that although transcript levels are predominantly regulated at the level of transcription, changes in mRNA stability play a role as well (Castells-Roca *et al.* 2011). We found that the majority (74%) of the genes induced at least 3-fold by heat shock in the study by Gasch *et al.* (2000) showed concordant increases in transcript abundance and in nascent RNA synthesis after heat shock, again highlighting the transcriptional activation that contributes to the increased RNA abundance for this class of genes. In addition, heat shock-inducible genes from the study by Gasch *et al.* 2000 showed a stronger correlation between changes in transcript abundance and changes in nascent transcript synthesis (Pearson’s *r^2^* = 0.55) compared with all yeast genes analyzed in this study (Pearson’s *r^2^* = 0.24). However, only 30 of the 304 genes with 2-fold or greater increased total RNA abundance after heat stress are known targets of Hsf1, a master transcriptional activator of the heat shock response, or contain Hsf1 or Msn2/4 recognition sites in their promoters (Table S7). Approximately 87% (26) of these 30 genes showed increased nascent transcript levels in response to heat shock. These findings suggest that additional factors may mediate stress-induced transcriptional activation.

## Discussion

In this study, we provide a high-resolution view of genome-wide transcriptional regulation in *S. cerevisiae*. By coupling the NRO assay to high-throughput sequencing, we assayed the synthesis of nascent RNA in yeast under normal growth conditions and in response to heat stress. We measured RNA abundance and synthesis in parallel, which revealed that cellular RNA abundance in yeast under both growth conditions is predominantly controlled by the rate of RNA synthesis. In accord with previous degradation experiments, we observed distinct functional gene classes between stable and unstable transcripts (Friedel *et al.* 2009; Grigull *et al.* 2004; Narsai *et al.* 2007; Wang *et al.* 2002).

These data allowed us to examine the spatial distribution of RNA synthesis along transcripts, which showed that nascent transcription in yeast is enriched near TSSs, as it is in humans. The data further suggest that postinitiation regulation in yeast may contribute to the control of gene expression (Churchman and Weissman 2011). Although recruitment of RNA polymerase to promoters appears to be rate-limiting for the large majority of yeast genes, transcription of up to 20% of yeast genes may be controlled at least in part at postinitiation steps. The mechanisms that determine polymerase pausing, arrest, or termination are not yet well understood. Many factors that modulate transcriptional pausing and the rate of elongation have been identified (Sims *et al.* 2004). Some of them are restricted to a subset of eukaryotes, with no homologs identified in yeast. However, in a recent study, Rodriguez-Gil *et al.* (2010) identified a subset of elongation-related factors (*e.g.* DSIF, Mediator, and the RNA polymerase II subunit Rpb9) that might influence the probability of RNA polymerase II pausing or arrest in yeast. In addition, the elongation factor TFIIS (yeast Dst1) stimulates intrinsic RNA polymerase RNA cleavage activity and thus promotes elongation of arrested RNA polymerase *in vivo* (Churchman and Weissman 2011). Signals in the DNA or RNA may affect RNA elongation; for example, both a hairpin structure in the nascent RNA and a run of uridines at the 3′ end of a transcript interfere with elongation and promote transcription termination (Bengal and Aloni 1989; Keene *et al.* 1999; Palangat *et al.* 1998).

The authors of recent studies have identified numerous cryptic unstable transcripts in yeast (Neil *et al.* 2009; Xu *et al.* 2009). Most of these transcripts were suggested to be byproducts of bidirectional transcription that originated from the same promoter but from distinct pre-initiation complexes (Neil *et al.* 2009). We observed little bidirectional transcription in either the NRO or total RNA samples. However, detection of cryptic unstable transcripts relied on the use of a strain defective in the nuclear exosome machinery (Neil *et al.* 2009; Xu *et al.* 2009). Thus, it is possible that most of the antisense transcription was undetectable in the strain used in our study, which is competent for RNA turnover. For nondivergent promoters, with an average intergenic distance of approximately 342 bp (Dujon 1996), a weak antisense signal arises at approximately −200 bp from the TSS, much upstream of the core promoter. Similarly, for divergent promoters, the antisense signal does not begin to accumulate until approximately −150 bp from the TSS. The distances at which antisense transcription is originated suggest that it likely results from the activity of a separate transcription pre-initiation complex assembled at a different core promoter.

An important biological question is how cells respond and cope with rapid changes in their environment, such as exposure to elevated temperatures (heat shock). Several genome-wide studies revealed that this response involves an increase in the abundance of transcripts from genes that are collectively termed “heat shock-inducible” (Gasch *et al.* 2000; Hahn *et al.* 2004). Earlier studies of the *Drosophila* hsp70 gene found that posttranscriptional regulation that affects RNA stability plays a role in the observed increase of hsp70 RNA levels in response to heat shock (Theodorakis and Morimoto 1987). Other studies of heat shock-inducible genes in *Drosophila* and humans found rapid transcriptional activation after heat shock, suggesting that this activation also contributes to increased RNA levels of heat shock-inducible genes (Mathur *et al.* 1994; O’Brien and Lis 1993). However, in previous studies investigators have not assessed global changes in nascent transcription in response to heat shock. Here, we found that both transcriptional activation and RNA stabilization likely play a role in the increased abundance of RNAs after heat shock treatment. Most of the heat shock-inducible genes (~74%) showed both an increase in nascent transcription and an increase in RNA abundance. Only approximately 14% of these genes increased their RNA abundance without increasing nascent transcription, indicative of RNA stabilization as the mechanism.

In summary, we used the NRO assay to provide a high-resolution view of transcriptional regulation in yeast. We applied this assay to examine the correlation between synthesis and abundance of transcripts, the distribution of polymerase activity along transcripts, and the genome-wide changes in yeast transcription in response to heat shock treatment. Further investigations may use similar approaches to characterize other modes of yeast transcriptional control under different growth conditions and in response to other environmental perturbations.

## Supplementary Material

Supporting Information
