# Supplementary material for: Genome-Wide Analysis of Nascent Transcription in Saccharomyces cerevisiae
Source: G3 (Bethesda). 2011 Dec 1;1(7):549–58. doi: 10.1534/g3.111.000810 (PMC3276176; doi:10.1534/g3.111.000810)
Supplement: Supporting Information [file supp_1_7_549__index.html]

Supporting Information 

# Genome-Wide Analysis of Nascent Transcription in *Saccharomyces cerevisiae*

## Supporting Information for McKinlay *et al.*, 2011

**Files in this Data Supplement:**

- Supporting Information - Figures S1-S10 and Tables S1-S7 (PDF, 2 MB)
- Figure S1 - Correlation between measurements of nascent transcription in yeast (PDF, 244 KB)
- Figure S2 - Genome-wide view of nascent transcription in yeast (PDF, 320 KB)
- Figure S3 - RNA polymerase II density is better correlated with nascent transcription than with transcript abundance (PDF, 192 KB)
- Figure S4 - Nascent transcripts are enriched for intronic sequences and show markedly less splicing activity (PDF, 116 KB)
- Figure S5 - The NRO to total RNA read depth ratio is drastically increased near TSSs (PDF, 100 KB)
- Figure S6 - NRO signal controls (PDF, 124 KB)
- Figure S7 - Distribution of genes in the 'active/not paused,' 'active/paused,' 'inactive/paused' and 'inactive/ not paused' categories as determined from NRO read densities in the promoter‐proximal 100 bp and body of the gene (≥101 bp downstream of TSS) (PDF, 260 KB)
- Figure S8 - Examples of read distribution along transcript models (PDF, 160 KB)
- Figure S9 - Antisense transcription in NRO and total RNA samples(PDF, 104 KB)
- Figure S10 - Correlation between estimated of yeast RNA stability (PDF, 232 KB)
- Table S1 - Specificity of enrichment of nascent biotinylated RNA on Streptavidin beads (PDF, 44 KB)
- Table S2 - Specificity of enrichment of *in vitro* synthesized biotinylated *Arabidopsis thaliana* RNA on Streptavidin beads (PDF, 52 KB)
- Table S3 - Sequencing data acquisition and mapping specifics (PDF, 44 KB)
- Table S4 - GO term enrichment analysis for analysis for transcripts at top of the ranking by ratios of nascent transcription to transcript abundance (GOrilla) (PDF, 96 KB)
- Table S5 - GO term enrichment analysis for transcripts at bottom of the ranking by ratios of nascent transcription to transcript abundance (GOrilla) (PDF, 172 KB)
- Table S6 - Sequencing data acquisition and mapping statistics of heatshock libraries (PDF, 32 KB)
- Table S7 - Heat shock induced genes highlight important roles for changes in transcription and stability (.xlsx, 92 KB)
