# Supplementary material for: Genome-Wide Analysis of Nascent Transcription in Saccharomyces cerevisiae
Source: G3 (Bethesda). 2011 Dec 1;1(7):549–58. doi: 10.1534/g3.111.000810 (PMC3276176; doi:10.1534/g3.111.000810)
Supplement: Supporting Information [file supp_1.7.549_000810SI.pdf]

Supporting Information

# Genome-wide Analysis of Nascent Transcription in Saccharomyces cerevisiae

Anastasia McKinlay*[1,§], Carlos L. Araya*[1,2], and Stanley Fields*[,§,§§]

* Department of Genome Sciences, University of Washington, Seattle, WA 98195, USA.
[§] Department of Medicine, University of Washington, Seattle, WA 98195, USA.
[§§] Howard Hughes Medical Institute.
[1] These authors contributed equally to this work.
[2] Present address: Department of Genetics, Stanford University, Stanford, CA 94301, USA.

## Supplementary References

1. Pelechano V, Jimeno-Gonzalez S, Rodriguez-Gil A, Garcia-Martinez J, Perez-Ortin JE, Chavez S: **Regulon-specific control of transcription elongation across the yeast genome**. *PLoS Genet* 2009, **5**(8):e1000614
2. Garcia-Martinez J, Aranda A, Perez-Ortin JE: **Genomic run-on evaluates transcription rates for all yeast genes and identifies gene regulatory mechanisms**. *Mol Cell* 2004, **15**(2):303-313.
3. Lefrancois P, Euskirchen GM, Auerbach RK, Rozowsky J, Gibson T, Yellman CM, Gerstein M, Snyder M: **Efficient yeast ChIP-Seq using multiplex short-read DNA sequencing**. *BMC Genomics* 2009, **10**:37.
4. Grigull J, Mnaimneh S, Pootoolal J, Robinson MD, Hughes TR: **Genome-wide analysis of mRNA stability using transcription inhibitors and microarrays reveals posttranscriptional control of ribosome biogenesis factors**. *Mol Cell Biol* 2004, **24**(12):5534-5547.
5. Core LJ, Waterfall JJ, Lis JT: **Nascent RNA sequencing reveals widespread pausing and divergent initiation at human promoters.** *Science* 2008, **322**(5909):1845-1848.
6. Wang Y, Liu CL, Storey JD, Tibshirani RJ, Herschlag D, Brown PO: **Precision and functional specificity in mRNA decay**. *Proc Natl Acad Sci U S A* 2002, **99**(9):5860-5865.
7. Gasch AP, Spellman PT, Kao CM, Carmel-Harel O, Eisen MB, Storz G, Botstein D, Brown PO: **Genomic expression programs in the response of yeast cells to environmental changes**. *Mol Biol Cell* 2000, **11**(12):4241-4257.

**Table S1. Specificity of enrichment of nascent biotinylated RNA on Streptavidin beads.**

| Gene | UTP (average Cp) | B16UTP (average Cp) | Fold enrichment (x) |
|---|---|---|---|
| **RDN18-1** | 17.82 | 13.26 | **23.6** |
| **ACT1** | 30.9 | 26.5 | **21.1** |
| **RPL28** | 31.13 | 27.99 | **8.9** |

Average Cp values correspond to the average cross-point values from triplicate qPCR reactions.

**Table S2. Specificity of enrichment of *in vitro* synthesized biotinylated *Arabidopsis thaliana* RNA on Streptavidin beads.**

| Transcript | UTP-RNA (Average Cp) | B16UTP-RNA (Average Cp) | Fold enrichment (x) |
|---|---|---|---|
| **ELF3 (779-926)** | 33.39 | 29.4 | 15.9 |
| **ELF3 (2631-2795)** | 37.8 | 32.52 | 38.8 |

*Average Cp values correspond to the average cross-point values from triplicate qPCR reactions*

**Table S3. Sequencing data acquisition and mapping statistics.**

| Library | Reads acquired | Reads mapped | Percent mapped | Unique, non-rRNA reads | Percent unique, non-rRNA reads |
|---|---|---|---|---|---|
| **NRO** | 63,688,617 | 55,409,215 | 87.00% | 2,492,414 | 4.50% |
| **RNA** | 83,607,712 | 83,148,829 | 99.45% | 939,582 | 1.13% |

**Table S4. GO term enrichment analysis for transcripts at top of the ranking by ratios of nascent transcription to transcript abundance (GOrilla).**

| | GO Term | Description | P-value | Enrichment | N | B | n | b |
|---|---|---|---|---|---|---|---|---|
| Process | GO:0015893 | drug transport | 5.11E-06 | 8.58 | 2383 | 13 | 171 | 8 |
| | GO:0006414 | translational elongation | 7.02E-06 | 4.85 | 2383 | 172 | 40 | 14 |
| | GO:0010033 | response to organic substance | 1.06E-05 | 1.83 | 2383 | 50 | 991 | 38 |
| | GO:0006855 | drug transmembrane transport | 1.25E-05 | 17.94 | 2383 | 8 | 83 | 5 |
| | GO:0000296 | spermine transport | 5.19E-05 | 238.3 | 2383 | 4 | 5 | 2 |
| | GO:0015849 | organic acid transport | 7.35E-05 | 2.55 | 2383 | 44 | 468 | 22 |
| | GO:0007165 | signal transduction | 1.00E-04 | 1.76 | 2383 | 117 | 578 | 50 |
| | GO:0030447 | filamentous growth | 1.51E-04 | 1.77 | 2383 | 50 | 940 | 35 |
| | GO:0006468 | protein amino acid phosphorylation | 1.55E-04 | 1.69 | 2383 | 64 | 923 | 42 |
| | GO:0006075 | 1,3-beta-glucan biosynthetic process | 1.64E-04 | 76.87 | 2383 | 2 | 31 | 2 |
| | GO:0006074 | 1,3-beta-glucan metabolic process | 1.64E-04 | 76.87 | 2383 | 2 | 31 | 2 |
| | GO:0051274 | beta-glucan biosynthetic process | 1.70E-04 | 38.44 | 2383 | 6 | 31 | 3 |
| | GO:0016310 | phosphorylation | 1.71E-04 | 1.65 | 2383 | 72 | 923 | 46 |
| | GO:0051273 | beta-glucan metabolic process | 3.35E-04 | 32.94 | 2383 | 7 | 31 | 3 |
| | GO:0019932 | second-messenger-mediated signaling | 3.58E-04 | 4.35 | 2383 | 15 | 329 | 9 |
| | GO:0016049 | cell growth | 4.10E-04 | 1.85 | 2383 | 37 | 940 | 27 |
| | GO:0015837 | amine transport | 4.31E-04 | 2.72 | 2383 | 30 | 468 | 16 |
| | GO:0055085 | transmembrane transport | 4.56E-04 | 1.55 | 2383 | 182 | 575 | 68 |
| | GO:0019236 | response to pheromone | 5.31E-04 | 2.16 | 2383 | 21 | 892 | 17 |
| | GO:0065009 | regulation of molecular function | 6.13E-04 | 2.51 | 2383 | 77 | 259 | 21 |
| | GO:0023052 | signaling | 6.69E-04 | 1.44 | 2383 | 137 | 909 | 75 |
| | GO:0023033 | signaling pathway | 6.69E-04 | 1.44 | 2383 | 137 | 909 | 75 |
| | GO:0015846 | polyamine transport | 7.73E-04 | 105.91 | 2383 | 9 | 5 | 2 |
| | GO:0070783 | growth of unicellular organism as a thread of | 8.37E-04 | 1.8 | 2383 | 38 | 940 | 27 |
| | GO:0044182 | filamentous growth of a population of unicellular | 8.37E-04 | 1.8 | 2383 | 38 | 940 | 27 |
| | GO:0030811 | regulation of nucleotide catabolic process | 9.57E-04 | 5.88 | 2383 | 21 | 135 | 7 |
| | GO:0033121 | regulation of purine nucleotide catabolic process | 9.57E-04 | 5.88 | 2383 | 21 | 135 | 7 |
| | GO:0007187 | G-protein signaling, coupled to cyclic nucleotide | 9.63E-04 | 7.24 | 2383 | 4 | 329 | 4 |
| | GO:0007188 | G-protein signaling, coupled to cAMP nucleotide | 9.63E-04 | 7.24 | 2383 | 4 | 329 | 4 |
| Function | GO:0004871 | signal transducer activity | 2.85E-07 | 2.76 | 2383 | 35 | 617 | 25 |
| | GO:0060089 | molecular transducer activity | 2.85E-07 | 2.76 | 2383 | 35 | 617 | 25 |
| | GO:0005215 | transporter activity | 8.30E-06 | 1.65 | 2383 | 180 | 611 | 76 |
| | GO:0004672 | protein kinase activity | 2.44E-05 | 1.78 | 2383 | 61 | 923 | 42 |
| | GO:0003700 | sequence-specific DNA binding transcription | 2.82E-05 | 1.71 | 2383 | 64 | 979 | 45 |
| | GO:0015297 | antiporter activity | 2.85E-05 | 74.47 | 2383 | 8 | 12 | 3 |
| | GO:0000297 | spermine transmembrane transporter activity | 5.19E-05 | 238.3 | 2383 | 4 | 5 | 2 |
| | GO:0022892 | substrate-specific transporter activity | 6.73E-05 | 1.65 | 2383 | 160 | 588 | 65 |
| | GO:0004674 | protein serine/threonine kinase activity | 6.77E-05 | 1.77 | 2383 | 57 | 923 | 39 |
| | GO:0022857 | transmembrane transporter activity | 7.08E-05 | 2.37 | 2383 | 153 | 197 | 30 |
| | GO:0005099 | Ras GTPase activator activity | 1.80E-04 | 7.27 | 2383 | 17 | 135 | 7 |
| | GO:0015238 | drug transmembrane transporter activity | 2.49E-04 | 11.96 | 2383 | 12 | 83 | 5 |
| | GO:0005096 | GTPase activator activity | 2.85E-04 | 5.3 | 2383 | 30 | 135 | 9 |
| | GO:0015203 | polyamine transmembrane transporter activity | 3.83E-04 | 136.17 | 2383 | 7 | 5 | 2 |
| | GO:0016773 | phosphotransferase activity, alcohol group as | 4.51E-04 | 1.55 | 2383 | 88 | 923 | 53 |
| | GO:0005088 | Ras guanyl-nucleotide exchange factor activity | 4.56E-04 | 4.56 | 2383 | 6 | 523 | 6 |
| | GO:0043565 | sequence-specific DNA binding | 4.63E-04 | 1.6 | 2383 | 120 | 697 | 56 |
| | GO:0015291 | secondary active transmembrane transporter | 5.99E-04 | 35.04 | 2383 | 17 | 12 | 3 |
| | GO:0005097 | Rab GTPase activator activity | 7.62E-04 | 8.83 | 2383 | 10 | 135 | 5 |
| | GO:0005275 | amine transmembrane transporter activity | 8.54E-04 | 2.53 | 2383 | 24 | 588 | 15 |
| Comp. | GO:0005886 | plasma membrane | 9.90E-07 | 3.95 | 2383 | 175 | 69 | 20 |
| | GO:0031224 | intrinsic to membrane | 3.57E-04 | 2.09 | 2383 | 460 | 72 | 29 |

*N: Total number of genes. B: Total number of genes with specific GO term (background). n: Total number of genes in "target" set. b: Total number of genes with specific GO term in "target" set.*

**Table S5. GO term enrichment analysis for transcripts at bottom of the ranking by ratios of nascent transcription to transcript abundance (GOrilla).**

| | GO Term | Description | P-value | Enrichment | N | B | n | b |
|---|---|---|---|---|---|---|---|---|
| Process | GO:0006412 | translation | 4.45E-09 | 1.59 | 2383 | 177 | 922 | 109 |
| | GO:0033044 | regulation of chromosome organization | 3.51E-04 | 38.23 | 2383 | 11 | 17 | 3 |
| | GO:0006334 | nucleosome assembly | 3.59E-04 | 5.02 | 2383 | 14 | 271 | 8 |
| | GO:0030004 | cellular monovalent inorganic cation homeostasis | 7.81E-04 | 4.79 | 2383 | 17 | 234 | 8 |
| Func. | GO:0003735 | structural constituent of ribosome | 6.38E-08 | 2.41 | 2383 | 119 | 366 | 44 |
| | GO:0005198 | structural molecule activity | 9.75E-06 | 1.89 | 2383 | 189 | 366 | 55 |
| Component | GO:0044444 | cytoplasmic part | 3.51E-07 | 1.14 | 2383 | 1190 | 984 | 559 |
| | GO:0044445 | cytosolic part | 4.82E-07 | 1.61 | 2383 | 136 | 905 | 83 |
| | GO:0033279 | ribosomal subunit | 7.64E-07 | 1.85 | 2383 | 126 | 623 | 61 |
| | GO:0032991 | macromolecular complex | 1.53E-06 | 1.45 | 2383 | 910 | 219 | 121 |
| | GO:0043228 | non-membrane-bounded organelle | 8.46E-06 | 1.29 | 2383 | 417 | 897 | 202 |
| | GO:0043232 | intracellular non-membrane-bounded organelle | 8.46E-06 | 1.29 | 2383 | 417 | 897 | 202 |
| | GO:0044422 | organelle part | 3.19E-05 | 1.16 | 2383 | 1177 | 641 | 368 |
| | GO:0044446 | intracellular organelle part | 3.19E-05 | 1.16 | 2383 | 1177 | 641 | 368 |
| | GO:0015935 | small ribosomal subunit | 3.79E-05 | 3.14 | 2383 | 54 | 267 | 19 |
| | GO:0022627 | cytosolic small ribosomal subunit | 4.48E-05 | 3.48 | 2383 | 41 | 267 | 16 |
| | GO:0044424 | intracellular part | 6.52E-05 | 1.05 | 2383 | 2149 | 688 | 649 |
| | GO:0005840 | ribosome | 7.19E-05 | 1.45 | 2383 | 175 | 875 | 93 |
| | GO:0030529 | ribonucleoprotein complex | 9.06E-05 | 1.31 | 2383 | 309 | 898 | 152 |
| | GO:0022625 | cytosolic large ribosomal subunit | 3.13E-04 | 1.74 | 2383 | 55 | 895 | 36 |
| | GO:0005737 | cytoplasm | 8.73E-04 | 1.12 | 2383 | 1034 | 884 | 430 |

*N: Total number of genes. B: Total number of genes with specific GO term (background). n: Total number of genes in "target" set. b: Total number of genes with specific GO term in "target" set.*

**Table S6. Sequencing data acquisition and mapping statistics of heatshock libraries.**

| Library | Reads acquired | Reads mapped | Percent mapped | Unique, non-rRNA reads | Percent unique, non-rRNA reads |
|---|---|---|---|---|---|
| **NRO (HS)** | 19,918,770 | 11,423,271 | 57.35% | 378,273 | 3.31% |
| **Total RNA (HS)** | 70,159,160 | 69,502,678 | 99.07% | 656,630 | 0.94% |

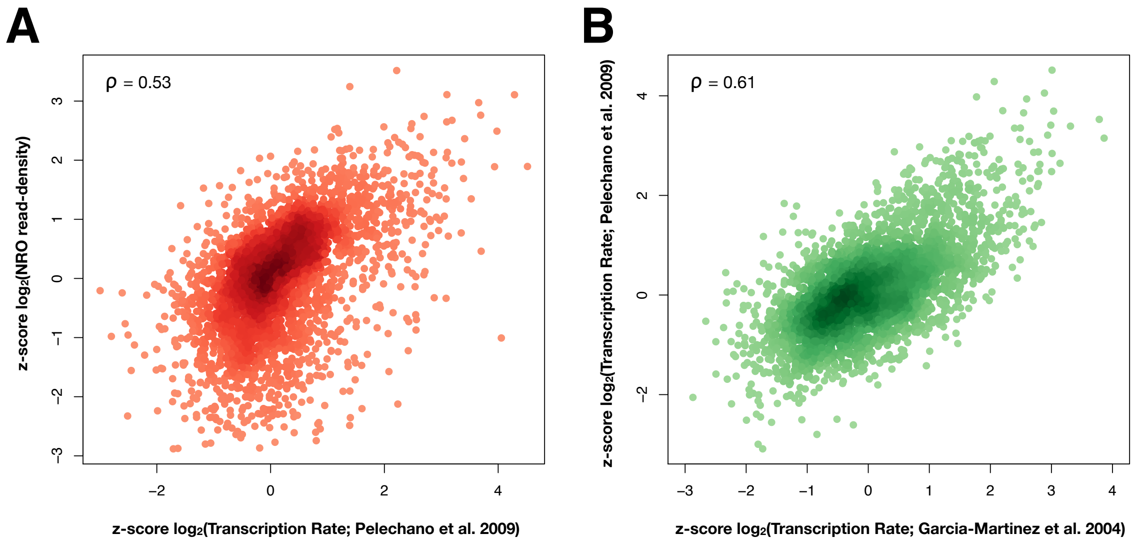


**Figure S1. Correlation between measurements of nascent transcription in yeast. (A)** Correlation between our NRO data and Pelechano et al. (2009) [1] estimates of transcription rate (Spearman's $\rho$ = 0.53). **(B)** Correlation between García-Martínez et al. (2004) [2] and Pelechano et al. (2009) [1] estimates of transcription rate (Spearman's $\rho$ = 0.61).

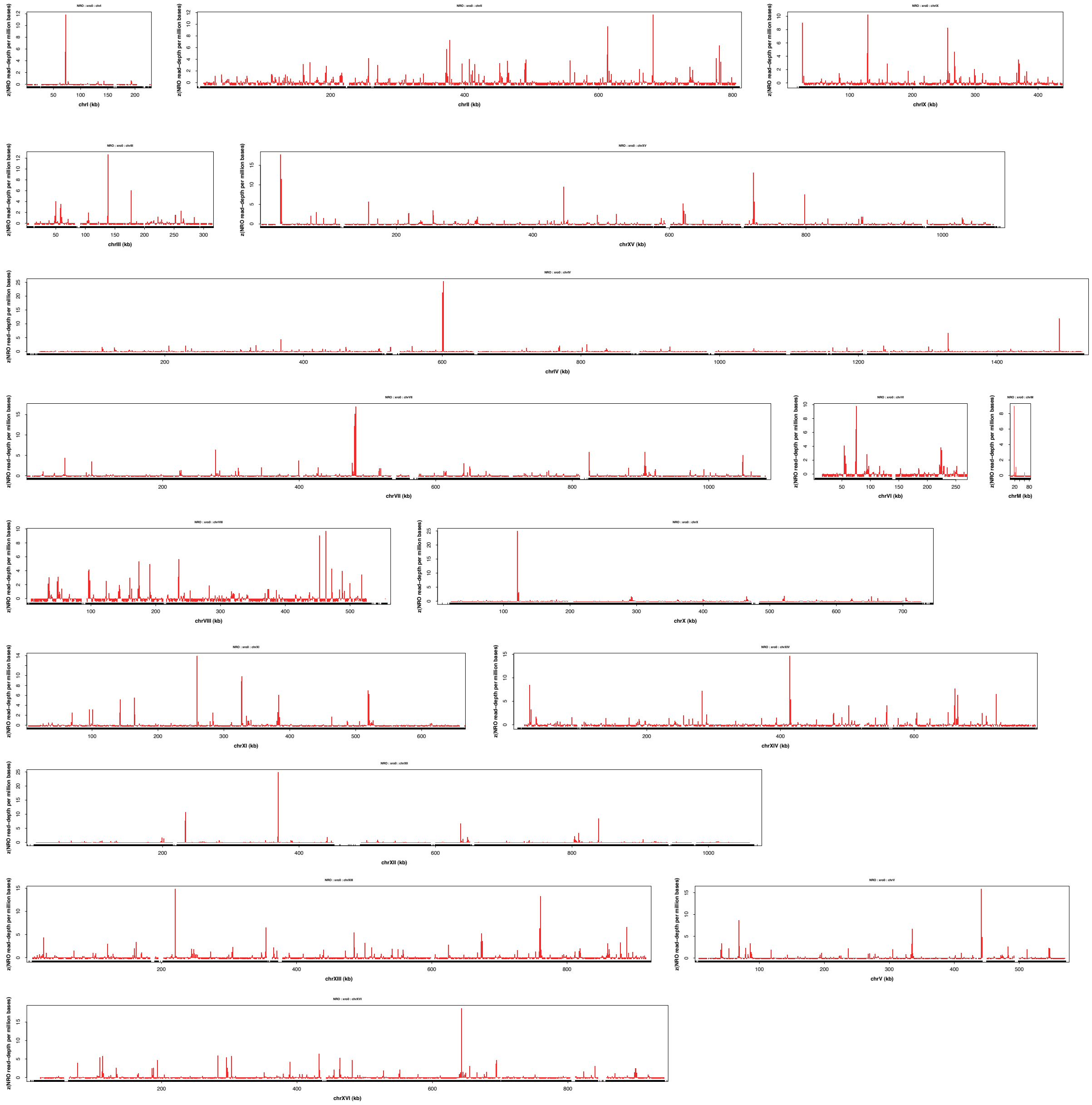


**Figure S2. Genome-wide view of nascent transcription in yeast.** *Z*-score of NRO read densities in 1 kb bins are shown for each chromosome. Genome-wide analysis of nascent synthesis revealed extensive transcription across the entire yeast genome. No > 3 kb clusters are observed to have read densities >3.1 *z*-scores.

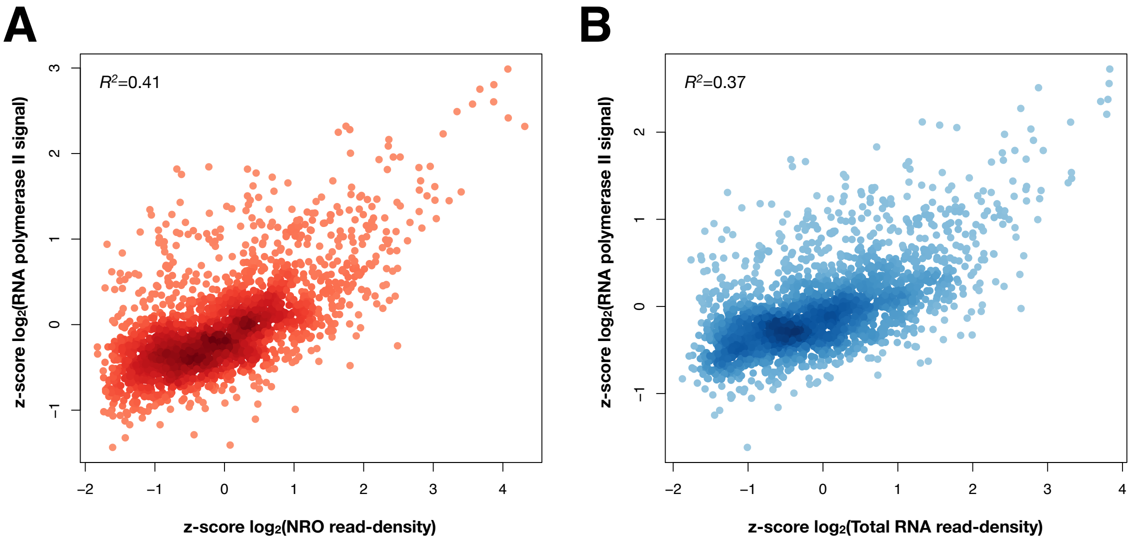


**Figure S3. RNA polymerase II density is better correlated with nascent transcription than with transcript abundance.** Correlation between NRO **(A)** and total RNA **(B)** read densities to RNA polymerase II density from Lefrancois et al. 2009 study [3] within non-overlapping transcript models (Pearson's $R^2$ = 0.41 and $R^2$ = 0.37, respectively).

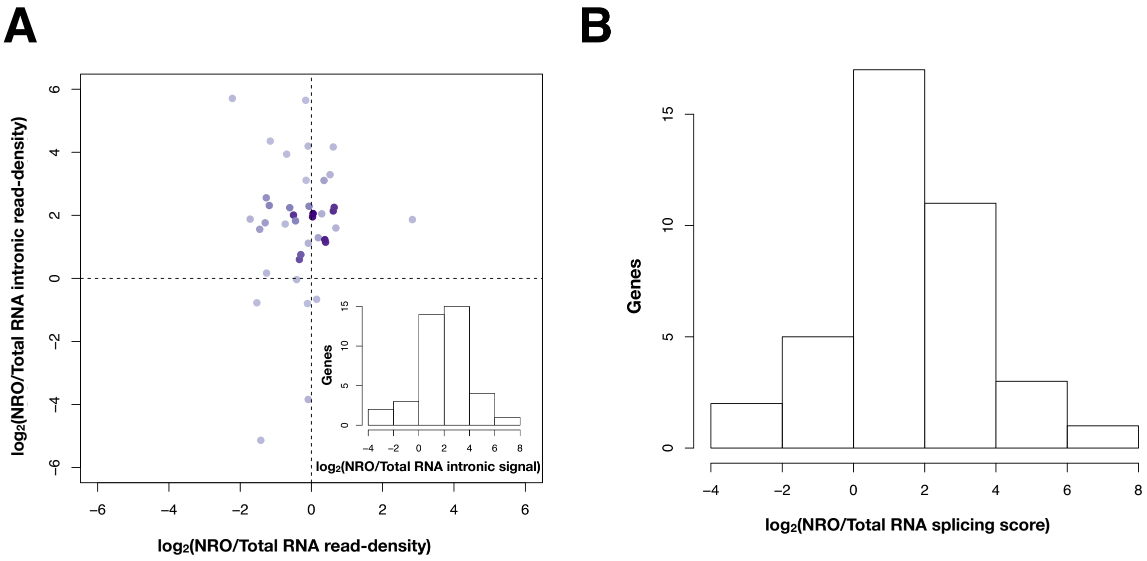


**Figure S4. Nascent transcripts are enriched for intronic sequences and show markedly less splicing activity.** **(A)** The log-transformed ratio of nascent and steady-state transcript levels (x-axis) is plotted against the log-transformed ratio of NRO and total RNA intronic read depths (y-axis) for 48 intron-containing transcripts. Inset: The ratio of intronic read densities in NRO and total RNA libraries was normalized by the fold difference in nascent and steady-state transcript levels. A histogram of these transcript-normalized intronic ratios is shown. **(B)** For each intron-containing gene (*N* = 48), a splicing score was calculated in NRO and total RNA libraries as the ratio of the mean intronic read depth over the mean read depth in flanking exons. As such, the splicing score measures the fraction of unspliced transcripts for each gene, where 1 indicates absence of splicing in the transcript and 0 indicates the transcript is fully spliced. To examine gains in unspliced transcript levels, a distribution of the ratios of NRO to total RNA splicing scores is shown.

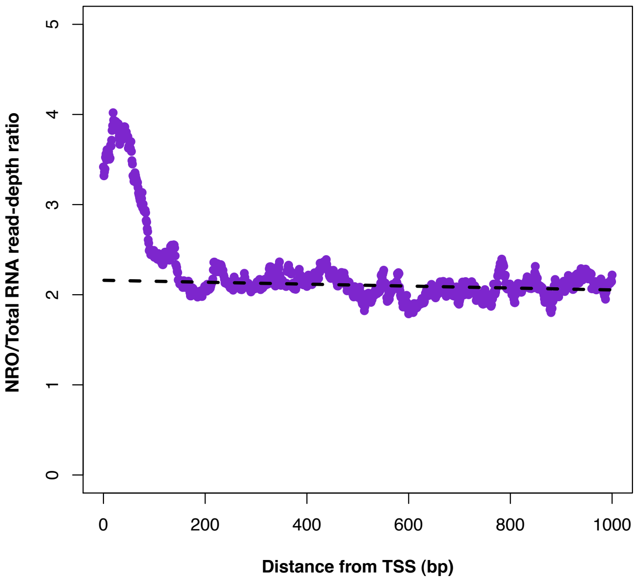


**Figure S5. The NRO to total RNA read depth ratio is drastically increased near TSSs.** The average ratio of NRO and total RNA read depth is plotted as a function of distance from TSSs for 2,530 genes that are transcriptionally active in both libraries. Dashed line represents the linear model regressed from positions 100-1000 bp. We observe a relatively constant ratio, beginning about 100 bp downstream of the TSS and continuing to the 3' end. However, this ratio was twice as high within the first 100 bp downstream of the TSS, suggesting that the enrichment of nascent transcription is specific to the promoter-proximal region.

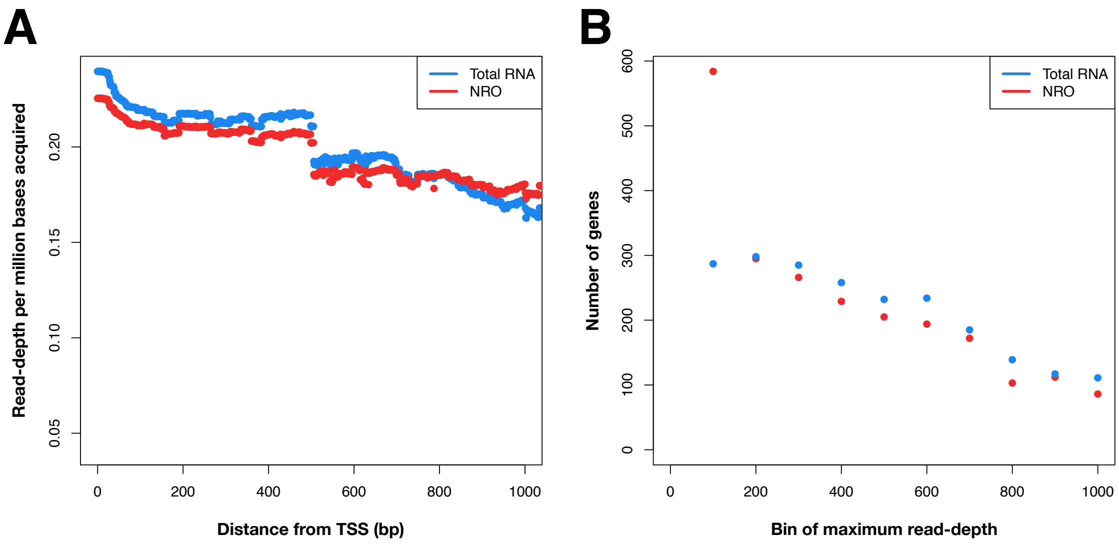


**Figure S6. NRO signal controls. (A)** We asked whether the promoter-proximal transcription peak could be the result of the increased average read density of short transcripts. We modeled the even distribution of reads within transcripts by computing the mean read depth for each transcript and plotted it as a function of distance from the TSS for the nascent and total RNA populations. This analysis showed similar profiles for the distribution of mean reads between nascent and total RNAs, suggesting that the peak of nascent transcription at the promoter-proximal region is not due to unequal sequencing coverage of transcripts of different length. **(B)** To rule out the possibility that the abundance of the promoter-proximal nascent RNA transcription is due to chance or to sequencing biases, we sorted transcripts into 100 bp-size bins corresponding to where their maximum read depth was observed. This analysis revealed that in nascent RNA libraries, maximum read depth was over two-fold more probable within the promoter-proximal 100 bp region than in any of the downstream 100 bp bins. Similarly, over twice as many genes in the nascent RNA library showed their maximum transcription in the first promoter-proximal bin compared to the total RNA library. In contrast, the maximum read depth was comparable between nascent and total RNAs in bins downstream of the promoter-proximal 100 bp. Thus, we conclude that the nascent transcription peak in the promoter-proximal region is not due to chance or sequencing biases.

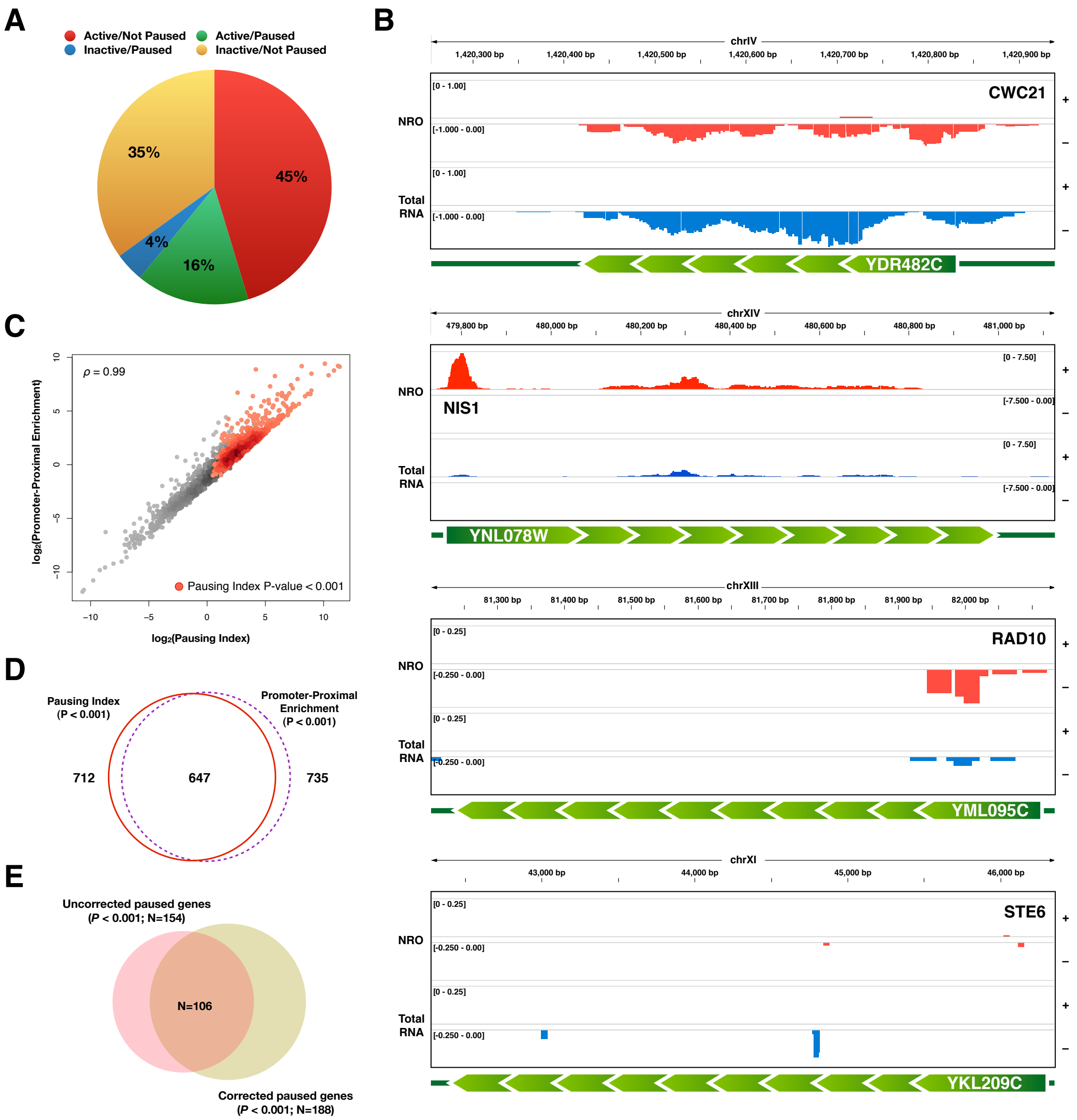


**Figure S7. (A)** Distribution of genes in the 'active/not paused,' 'active/paused,' 'inactive/paused' and 'inactive/not paused' categories as determined from NRO read densities in the promoter-proximal 100 bp and body of the gene (≥101 bp downstream of TSS). To identify genes with promoter-proximal enrichment of RNA polymerase II activity we applied a strategy similar to the one described by Core et al. (2008), which allows a classification of genes by pausing and activity. We calculated for each gene a pausing index consisting of the ratio of read density in the promoter-proximal region (the 100 bp downstream of the TSS) relative to that in the body of the gene (from 101 bp downstream of the TSS to the termination site). We classified genes as 'paused' if the NRO read density within the promoter-proximal region was significantly higher than in the body of the gene ($P < 0.001$), or 'not paused' if this condition was not met. For both 'paused' and 'not paused' genes, a gene was considered 'active' if the NRO read density from 201 bp downstream of the TSS was significant ($P < 0.01$), or 'inactive' if it was not. **(B)** Examples of genes classified by pausing versus activity. IGV genome browser views (http://www.broadinstitute.org/igv) of NRO (red) and total RNA (blue) read density in transcript models for prototypical genes classified as 'active/not paused' (*CWC21*), 'active/paused' (*NIS1*), 'inactive/paused' (*RAD10*), and 'inactive/not paused' (*STE6*). With the exception of *RAD10*, the region shown encompasses the transcript model (from TSS to termination site). Normalized read depth is shown in the plus and minus strands as positive and negative values, respectively. Read depth range is indicated between brackets. Transcript models are schematized below each gene with arrows in green indicating the coding region of each gene. **(C)** Correlation between the modeled promoter-proximal enrichment and pausing

indices. For each gene, read depth from 101 bp downstream of the TSS was used to generate a linear model of read depth throughout the transcript. Expected read density in the promoter-proximal 100 bp was extrapolated from linear models with natural intersects. A promoter-proximal enrichment ratio of the observed over the predicted read density was then estimated and compared to the pausing indices reported in this study. The correlation between promoter-proximal enrichment and pausing indices is shown for 2,578 genes with significant read densities in NRO and total RNA libraries, grey dots. Transcripts with statistically significant pausing indices ($P < 0.001$) are highlighted in red. The significance of promoter-proximal enrichment was determined by testing for significance of the observed read depths against the expected values using a Poisson test. **(D)** The overlap between genes with significant ($P < 0.001$) promoter-proximal enrichments ($N =$ 735, dotted purple circle) and genes with significant ($P < 0.001$) pausing indices ($N = 712$, solid red line) is shown. **(E)** Sequencing bias does not drive pausing classification. To examine the effect of sequencing biases on the analysis of pausing, we examined sequencing bias in genes with steady-state transcript levels two-fold above the mean (N = 414). In this set of high-abundance transcripts, we calculated the sequencing bias as the adjustment ratio (ψ) of total RNA library read density in the promoter-proximal 100 bp and the gene body. NRO read density in the promoter-proximal 100 bp was normalized by the derived, transcript-specific adjustment ratio (ψ) for each gene. Transcripts were then analyzed for pausing using the Poisson test described above following corrections. ~70% of the genes determined to be paused in the standard ('uncorrected') analysis remain classified as paused after read density adjustment ('corrected') in the NRO libraries.

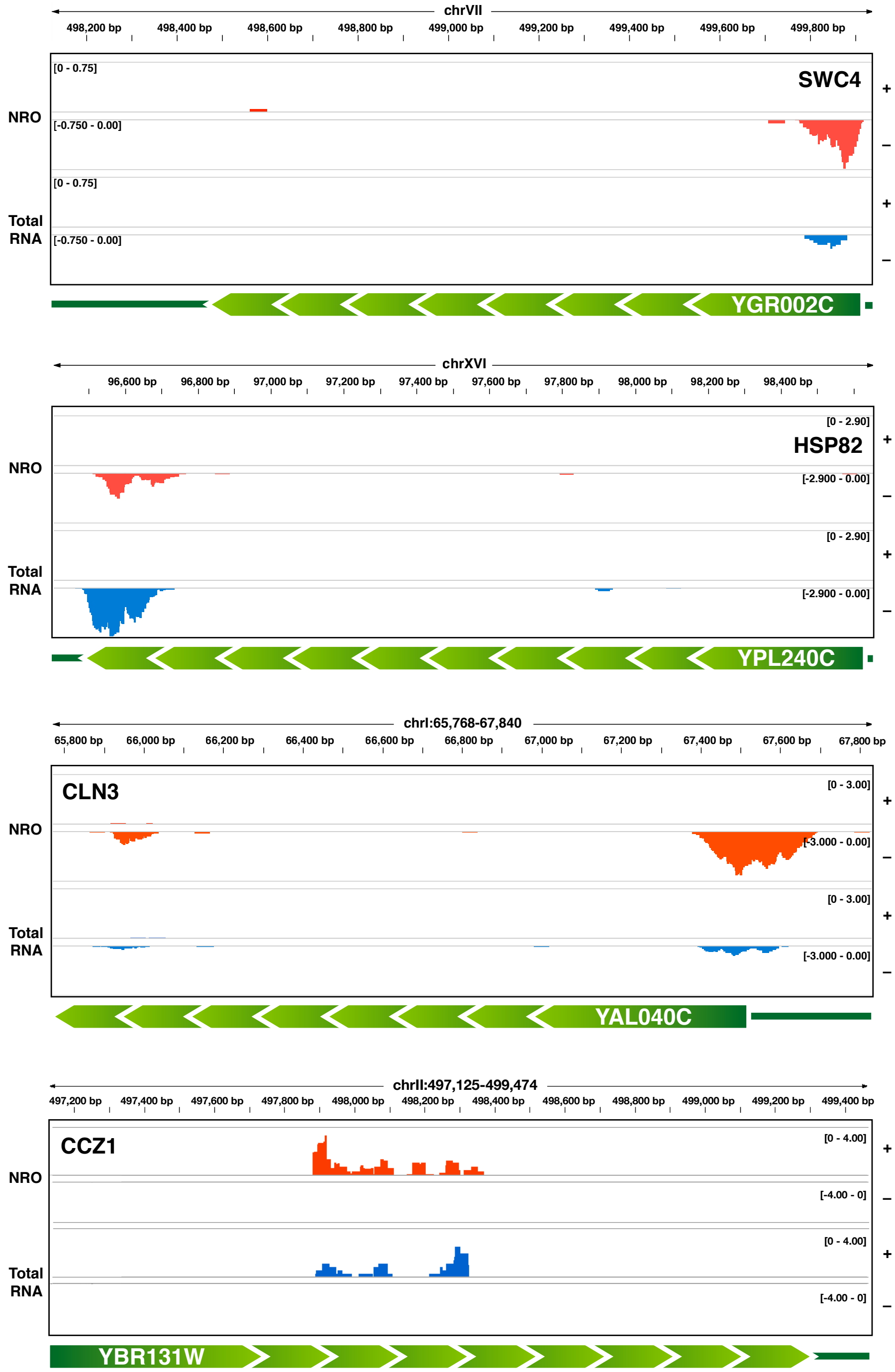


**Figure S8. Examples of read distribution along transcript models.** NRO (red) and total RNA (blue) read densities are shown for genes with 5' accumulated read depths (*SWC4*), the 3' accumulated read depths (*HSP82*), transcripts with read depth peaks at both promoter-proximal region and near transcription termination site (*CLN3*) and for transcripts whose peak of nascent transcription occurs near the middle of the gene (*CCZ1*). For all transcripts, normalized read depth is shown in the plus and minus strands as positive and negative values, respectively. Read depth range is indicated between brackets. Transcript models are schematized below each gene with arrows in green indicating the coding region of each gene.

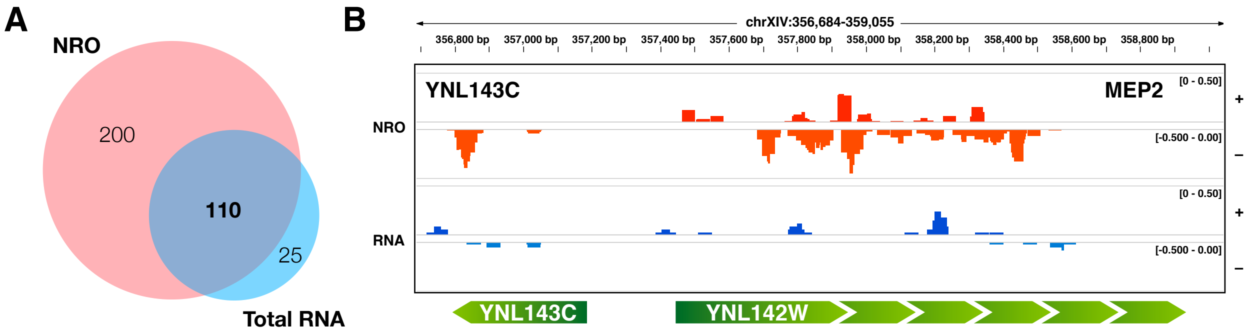


**Figure S9. Antisense transcription in NRO and total RNA samples. (A)** Venn diagram showing number of transcript models with significant antisense transcription in NRO (red) and total RNA (blue) samples and their overlap. As for expression analysis, the significance of antisense transcription within these models was calculated against the Poisson distribution of read depth per base expected from the background RNA-seq read depth in intergenic regions. A *P*-value significance cutoff of 0.01 was applied. **(B)** Prevalence of antisense transcription in NRO sample is exemplified by read depth in the *MEP2* gene.

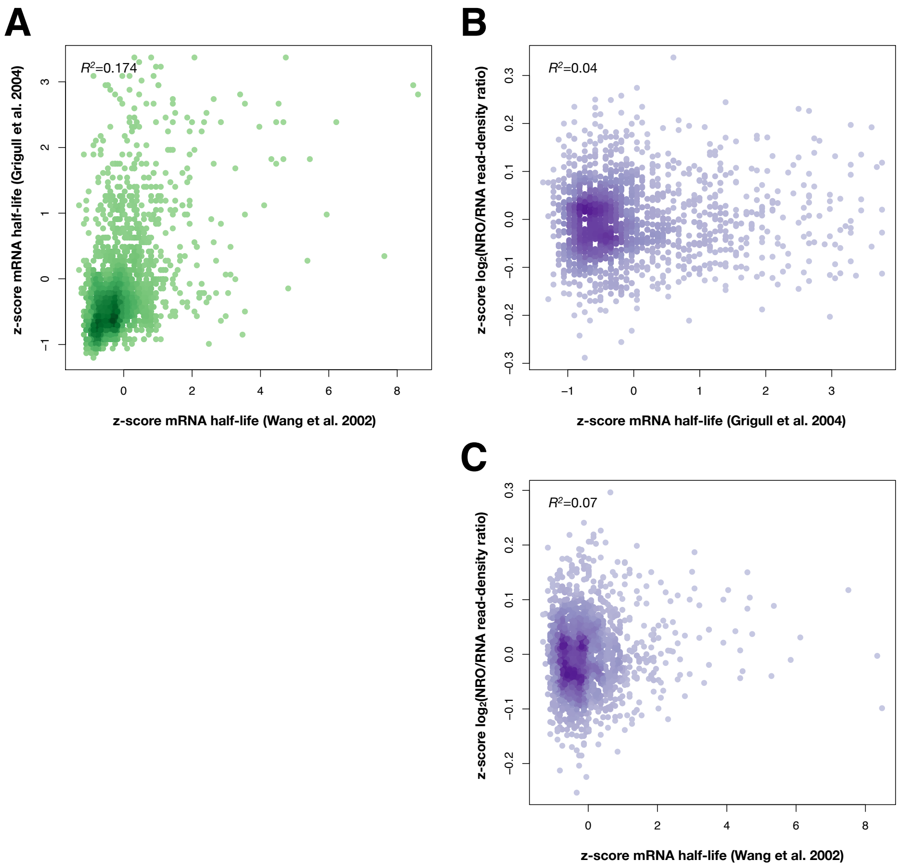


**Figure S10. Correlation between estimates of yeast RNA stability. (A)** Correlation between mRNA half-life measurements by Grigull et al. (2004) [4] and Wang et al. (2002) [5] (Pearson's $R^2$ = 0.174). **(B, C)** Correlations between our calculated nascent transcript stability and Grigull et al. (2004) [4] **(B)** or Wang et al. (2002) [5] **(C)** data sets (Pearson's $R^2$ = 0.04 and $R^2$ = 0.07, respectively).
