# Supplementary material for: Genome-Wide Analysis of Nascent Transcription in Saccharomyces cerevisiae
Source: G3 (Bethesda). 2011 Dec 1;1(7):549–58. doi: 10.1534/g3.111.000810 (PMC3276176; doi:10.1534/g3.111.000810)
Supplement: Supporting Information [file supp_1.7.549_FigureS1.pdf]

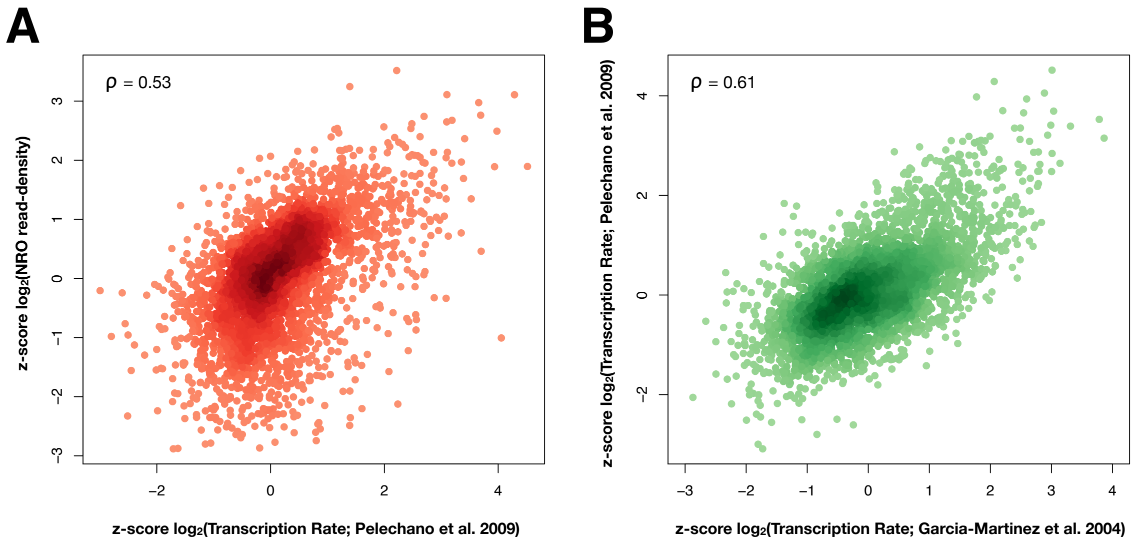

Figure S1. Correlation between measurements of nascent transcription in yeast. (A) Correlation between our NRO data and Pelechano et al. (2009) [1] estimates of transcription rate (Spearman's ρ = 0.53). **(B)** Correlation between García-Martínez et al. (2004) [2] and Pelechano et al. (2009) [1] estimates of transcription rate (Spearman's ρ = 0.61).
