# Supplementary material for: Genome-Wide Analysis of Nascent Transcription in Saccharomyces cerevisiae
Source: G3 (Bethesda). 2011 Dec 1;1(7):549–58. doi: 10.1534/g3.111.000810 (PMC3276176; doi:10.1534/g3.111.000810)
Supplement: Supporting Information [file supp_1.7.549_FigureS2.pdf]

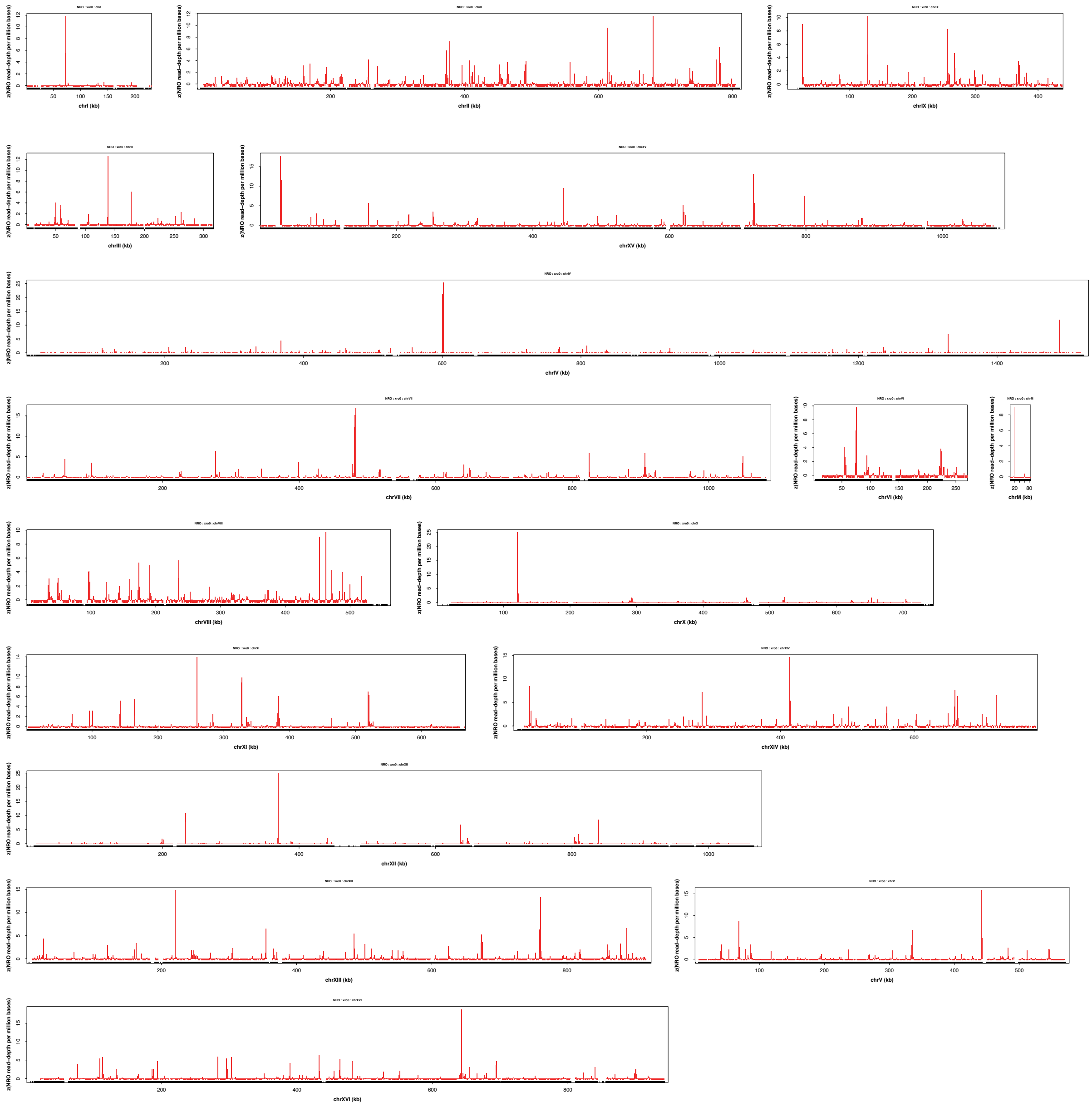

Figure S2. Genome-wide view of nascent transcription in yeast. *Z*-score of NRO read densities in 1 kb bins are shown for each chromosome. Genome-wide analysis of nascent synthesis revealed extensive transcription across the entire yeast genome. No > 3 kb clusters are observed to have read densities >3.1 *z*-scores.
