# Supplementary material for: Genome-Wide Analysis of Nascent Transcription in Saccharomyces cerevisiae
Source: G3 (Bethesda). 2011 Dec 1;1(7):549–58. doi: 10.1534/g3.111.000810 (PMC3276176; doi:10.1534/g3.111.000810)
Supplement: Supporting Information [file supp_1.7.549_FigureS3.pdf]

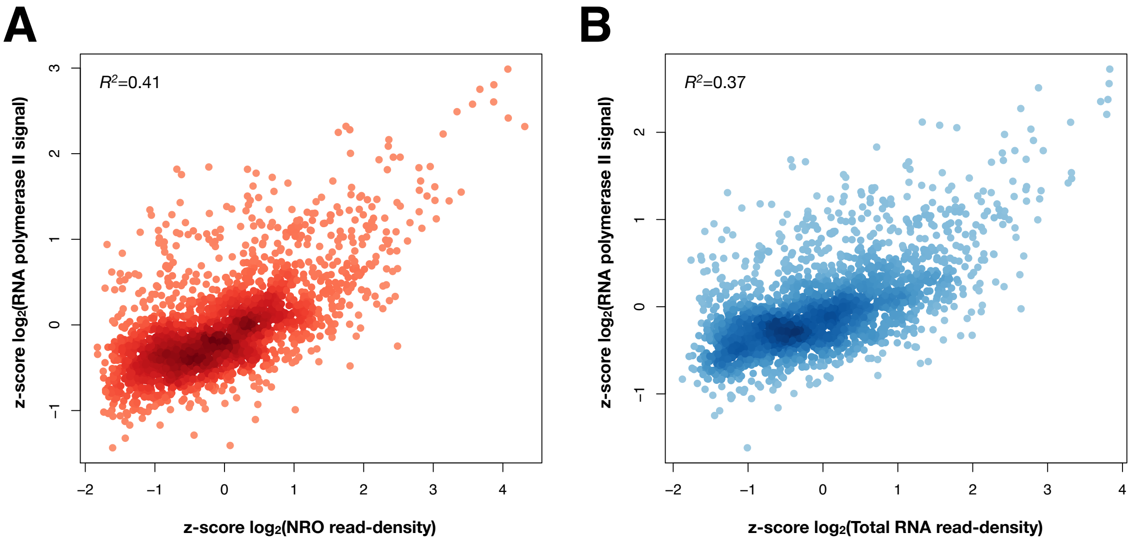

Figure S3. RNA polymerase II density is better correlated with nascent transcription than with transcript abundance. Correlation between NRO **(A)** and total RNA **(B)** read densities to RNA polymerase II density from Lefrancois et al. 2009 study [3] within non-overlapping transcript models (Pearson's R^2 = 0.41 and R^2 = 0.37, respectively).
