# Supplementary material for: Genome-Wide Analysis of Nascent Transcription in Saccharomyces cerevisiae
Source: G3 (Bethesda). 2011 Dec 1;1(7):549–58. doi: 10.1534/g3.111.000810 (PMC3276176; doi:10.1534/g3.111.000810)
Supplement: Supporting Information [file supp_1.7.549_FigureS4.pdf]

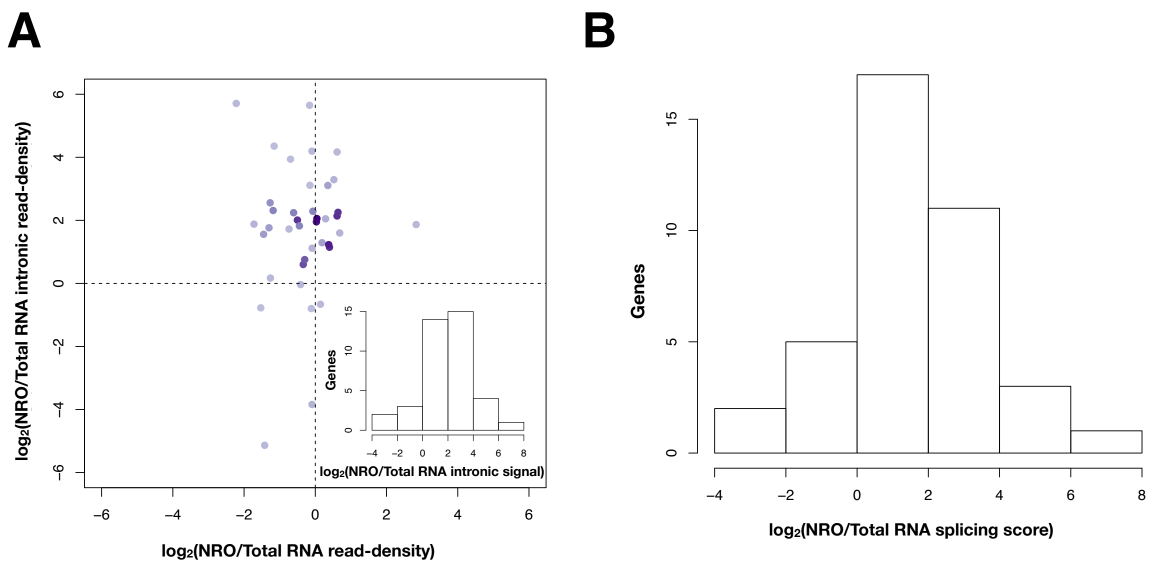

Figure S4. Nascent transcripts are enriched for intronic sequences and show markedly less splicing activity. (A) The log-transformed ratio of nascent and steady-state transcript levels (x-axis) is plotted against the log-transformed ratio of NRO and total RNA intronic read depths (y-axis) for 48 intron-containing transcripts. Inset: The ratio of intronic read densities in NRO and total RNA libraries was normalized by the fold difference in nascent and steady-state transcript levels. A histogram of these transcript-normalized intronic ratios is shown. **(B)** For each intron-containing gene (*N* = 48), a splicing score was calculated in NRO and total RNA libraries as the ratio of the mean intronic read depth over the mean read depth in flanking exons. As such, the splicing score measures the fraction of unspliced transcripts for each gene, where 1 indicates absence of splicing in the transcript and 0 indicates the transcript is fully spliced. To examine gains in unspliced transcript levels, a distribution of the ratios of NRO to total RNA splicing scores is shown.
