# Supplementary material for: Genome-Wide Analysis of Nascent Transcription in Saccharomyces cerevisiae
Source: G3 (Bethesda). 2011 Dec 1;1(7):549–58. doi: 10.1534/g3.111.000810 (PMC3276176; doi:10.1534/g3.111.000810)
Supplement: Supporting Information [file supp_1.7.549_FigureS5.pdf]

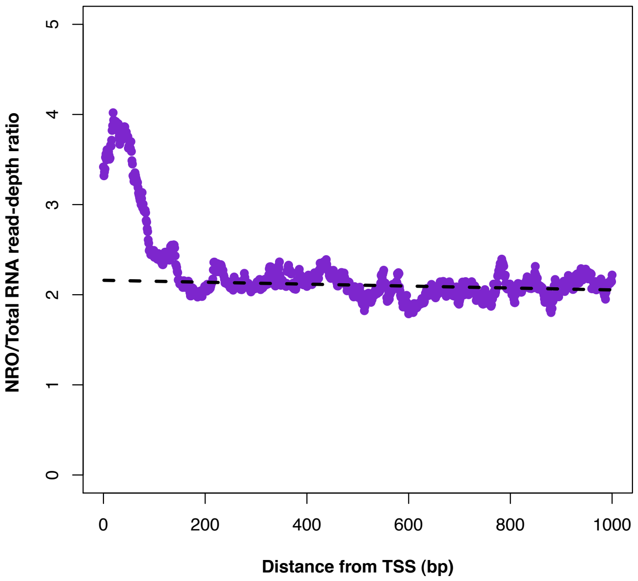

Figure S5. The NRO to total RNA read depth ratio is drastically increased near TSSs. The average ratio of NRO and total RNA read depth is plotted as a function of distance from TSSs for 2,530 genes that are transcriptionally active in both libraries. Dashed line represents the linear model regressed from positions 100-1000 bp. We observe a relatively constant ratio, beginning about 100 bp downstream of the TSS and continuing to the 3' end. However, this ratio was twice as high within the first 100 bp downstream of the TSS, suggesting that the enrichment of nascent transcription is specific to the promoter-proximal region.
