# Supplementary material for: Genome-Wide Analysis of Nascent Transcription in Saccharomyces cerevisiae
Source: G3 (Bethesda). 2011 Dec 1;1(7):549–58. doi: 10.1534/g3.111.000810 (PMC3276176; doi:10.1534/g3.111.000810)
Supplement: Supporting Information [file supp_1.7.549_FigureS6.pdf]

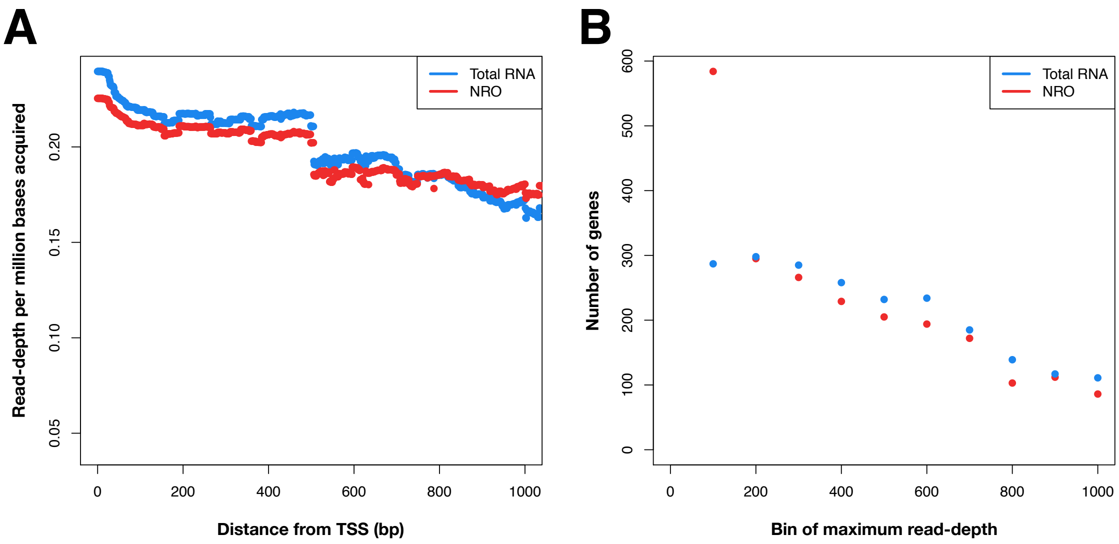

Figure S6. NRO signal controls. (A) We asked whether the promoter-proximal transcription peak could be the result of the increased average read density of short transcripts. We modeled the even distribution of reads within transcripts by computing the mean read depth for each transcript and plotted it as a function of distance from the TSS for the nascent and total RNA populations. This analysis showed similar profiles for the distribution of mean reads between nascent and total RNAs, suggesting that the peak of nascent transcription at the promoter-proximal region is not due to unequal sequencing coverage of transcripts of different length. **(B)** To rule out the possibility that the abundance of the promoter-proximal nascent RNA transcription is due to chance or to sequencing biases, we sorted transcripts into 100 bp-size bins corresponding to where their maximum read depth was observed. This analysis revealed that in nascent RNA libraries, maximum read depth was over two-fold more probable within the promoter-proximal 100 bp region than in any of the downstream 100 bp bins. Similarly, over twice as many genes in the nascent RNA library showed their maximum transcription in the first promoter-proximal bin compared to the total RNA library. In contrast, the maximum read depth was comparable between nascent and total RNAs in bins downstream of the promoter-proximal 100 bp. Thus, we conclude that the nascent transcription peak in the promoter-proximal region is not due to chance or sequencing biases.
