# Supplementary material for: Genome-Wide Analysis of Nascent Transcription in Saccharomyces cerevisiae
Source: G3 (Bethesda). 2011 Dec 1;1(7):549–58. doi: 10.1534/g3.111.000810 (PMC3276176; doi:10.1534/g3.111.000810)
Supplement: Supporting Information [file supp_1.7.549_FigureS7.pdf]

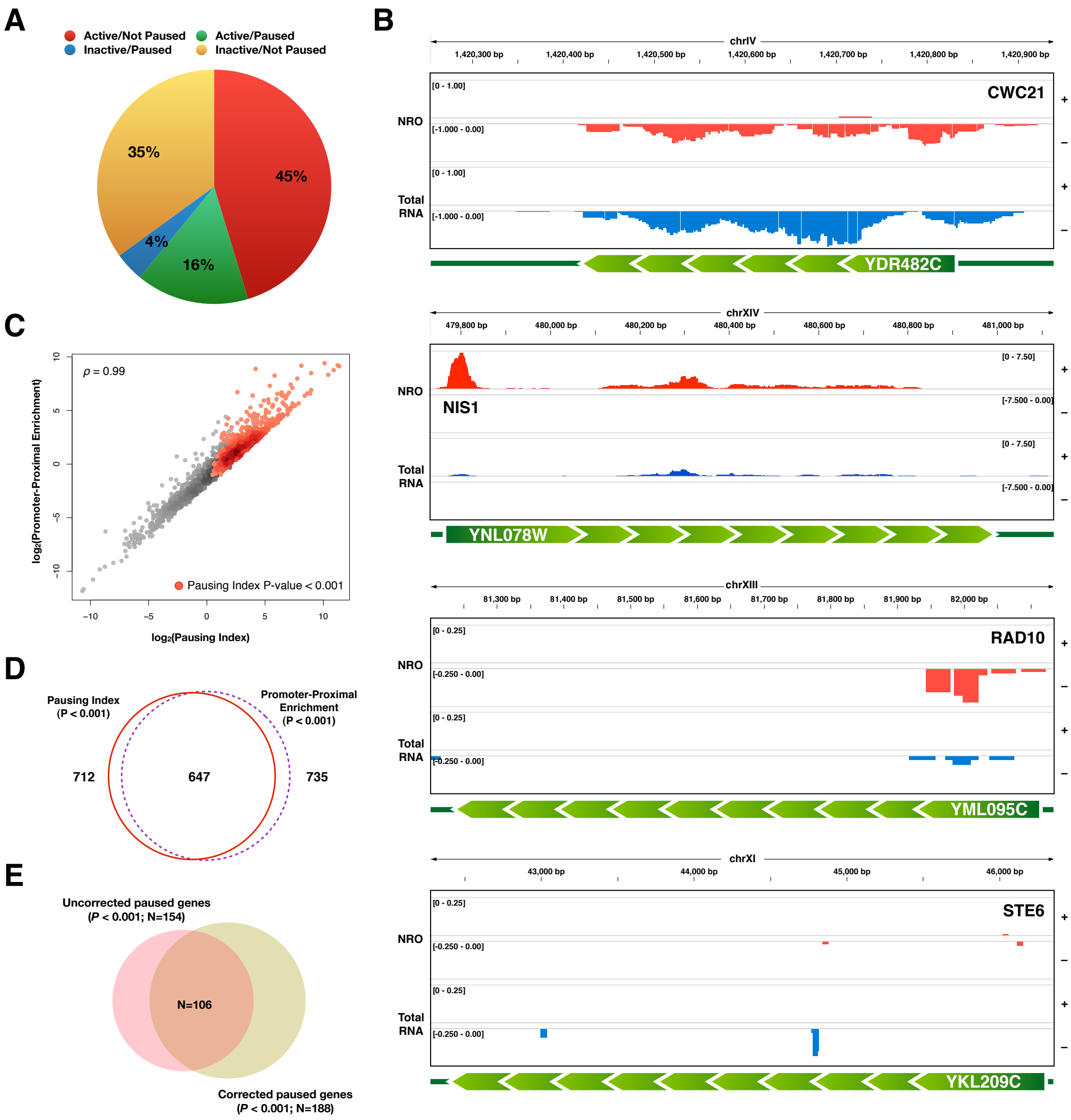

Figure S7. (A) Distribution of genes in the 'active/not paused,' 'active/paused,' 'inactive/paused' and 'inactive/not paused' categories as determined from NRO read densities in the promoter-proximal 100 bp and body of the gene (≥101 bp downstream of TSS). To identify genes with promoter-proximal enrichment of RNA polymerase II activity we applied a strategy similar to the one described by Core et al. (2008), which allows a classification of genes by pausing and activity. We calculated for each gene a pausing index consisting of the ratio of read density in the promoter-proximal region (the 100 bp downstream of the TSS) relative to that in the body of the gene (from 101 bp downstream of the TSS to the termination site). We classified genes as 'paused' if the NRO read density within the promoter-proximal region was significantly higher than in the body of the gene ($P < 0.001$), or 'not paused' if this condition was not met. For both 'paused' and 'not paused' genes, a gene was considered 'active' if the NRO read density from 201 bp downstream of the TSS was significant ($P < 0.01$), or 'inactive' if it was not. **(B)** Examples of genes classified by pausing versus activity. IGV genome browser views (http://www.broadinstitute.org/igv) of NRO (red) and total RNA (blue) read density in transcript models for prototypical genes classified as 'active/not paused' (*CWC21*), 'active/paused' (*NIS1*), 'inactive/paused' (*RAD10*), and 'inactive/not paused' (*STE6*). With the exception of *RAD10*, the region shown encompasses the transcript model (from TSS to termination site). Normalized read depth is shown in the plus and minus strands as positive and negative values, respectively. Read depth range is indicated between brackets. Transcript models are schematized below each gene with arrows in green indicating the coding region of each gene. **(C)** Correlation between the modeled promoter-proximal enrichment and pausing

indices. For each gene, read depth from 101 bp downstream of the TSS was used to generate a linear model of read depth throughout the transcript. Expected read density in the promoter-proximal 100 bp was extrapolated from linear models with natural intersects. A promoter-proximal enrichment ratio of the observed over the predicted read density was then estimated and compared to the pausing indices reported in this study. The correlation between promoter-proximal enrichment and pausing indices is shown for 2,578 genes with significant read densities in NRO and total RNA libraries, grey dots. Transcripts with statistically significant pausing indices ($P < 0.001$) are highlighted in red. The significance of promoter-proximal enrichment was determined by testing for significance of the observed read depths against the expected values using a Poisson test. **(D)** The overlap between genes with significant ($P < 0.001$) promoter-proximal enrichments ($N = 735$, dotted purple circle) and genes with significant ($P < 0.001$) pausing indices ($N = 712$, solid red line) is shown. **(E)** Sequencing bias does not drive pausing classification. To examine the effect of sequencing biases on the analysis of pausing, we examined sequencing bias in genes with steady-state transcript levels two-fold above the mean (N = 414). In this set of high-abundance transcripts, we calculated the sequencing bias as the adjustment ratio (ψ) of total RNA library read density in the promoter-proximal 100 bp and the gene body. NRO read density in the promoter-proximal 100 bp was normalized by the derived, transcript-specific adjustment ratio (ψ) for each gene. Transcripts were then analyzed for pausing using the Poisson test described above following corrections. ~70% of the genes determined to be paused in the standard ('uncorrected') analysis remain classified as paused after read density adjustment ('corrected') in the NRO libraries.
