# Supplementary material for: Genome-Wide Analysis of Nascent Transcription in Saccharomyces cerevisiae
Source: G3 (Bethesda). 2011 Dec 1;1(7):549–58. doi: 10.1534/g3.111.000810 (PMC3276176; doi:10.1534/g3.111.000810)
Supplement: Supporting Information [file supp_1.7.549_FigureS8.pdf]

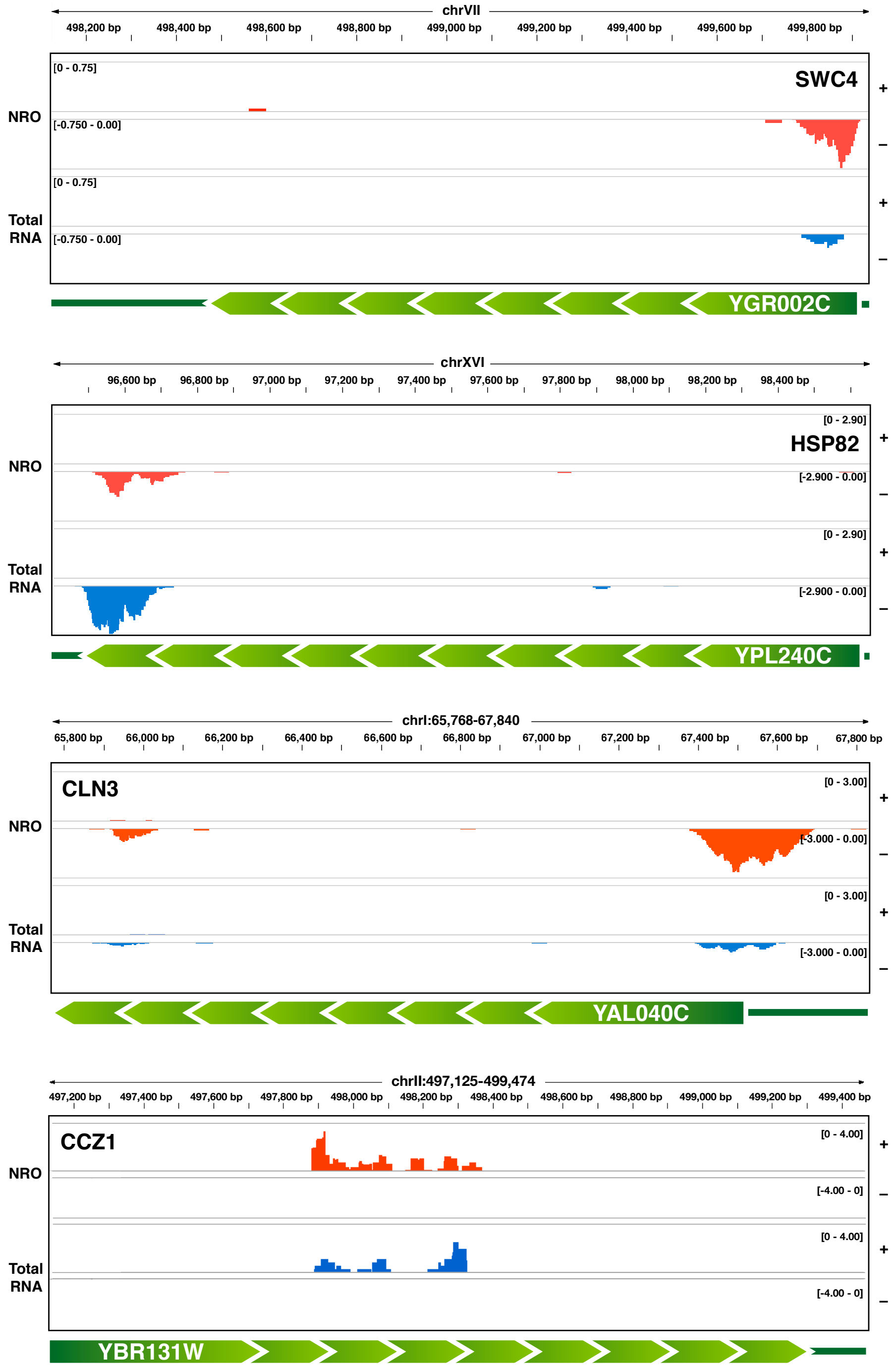

Figure S8. Examples of read distribution along transcript models. NRO (red) and total RNA (blue) read densities are shown for genes with 5' accumulated read depths (*SWC4*), the 3' accumulated read depths (*HSP82*), transcripts with read depth peaks at both promoter-proximal region and near transcription termination site (*CLN3*) and for transcripts whose peak of nascent transcription occurs near the middle of the gene (*CCZ1*). For all transcripts, normalized read depth is shown in the plus and minus strands as positive and negative values, respectively. Read depth range is indicated between brackets. Transcript models are schematized below each gene with arrows in green indicating the coding region of each gene.
