# Supplementary material for: Genome-Wide Analysis of Nascent Transcription in Saccharomyces cerevisiae
Source: G3 (Bethesda). 2011 Dec 1;1(7):549–58. doi: 10.1534/g3.111.000810 (PMC3276176; doi:10.1534/g3.111.000810)
Supplement: Supporting Information [file supp_1.7.549_FigureS10.pdf]

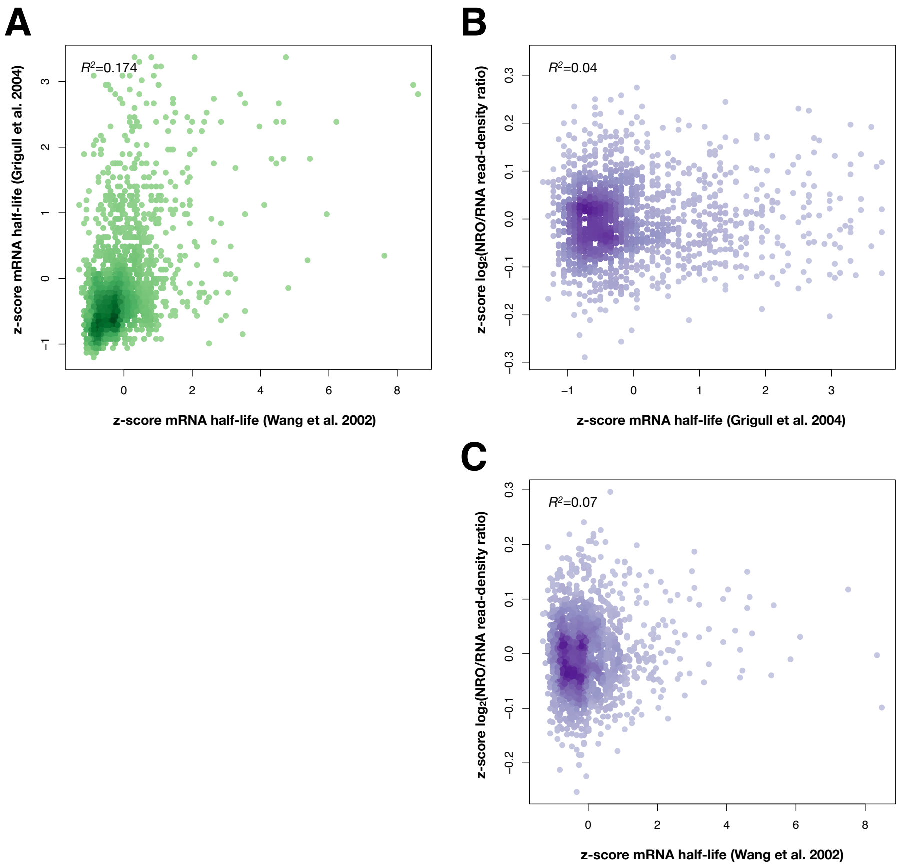

Figure S10. Correlation between estimates of yeast RNA stability. (A) Correlation between mRNA half-life measurements by Grigull et al. (2004) [4] and Wang et al. (2002) [5] (Pearson's R^2 = 0.174). **(B, C)** Correlations between our calculated nascent transcript stability and Grigull et al. (2004) [4] **(B)** or Wang et al. (2002) [5] **(C)** data sets (Pearson's R^2 = 0.04 and R^2 = 0.07, respectively).
