# Supplementary material for: Genome-Wide Analysis of Nascent Transcription in Saccharomyces cerevisiae
Source: G3 (Bethesda). 2011 Dec 1;1(7):549–58. doi: 10.1534/g3.111.000810 (PMC3276176; doi:10.1534/g3.111.000810)
Supplement: Supporting Information [file supp_1.7.549_TableS1.pdf]

Table S1. Specificity of enrichment of nascent biotinylated RNA on Streptavidin beads.

Gene	UTP (average Cp)	B16UTP (average Cp)	Fold enrichment (x)
**RDN18-1**	17.82	13.26	**23.6**
**ACT1**	30.9	26.5	**21.1**
**RPL28**	31.13	27.99	**8.9**

Average Cp values correspond to the average cross-point values from triplicate qPCR reactions.
