# Supplementary material for: Genome-Wide Analysis of Nascent Transcription in Saccharomyces cerevisiae
Source: G3 (Bethesda). 2011 Dec 1;1(7):549–58. doi: 10.1534/g3.111.000810 (PMC3276176; doi:10.1534/g3.111.000810)
Supplement: Supporting Information [file supp_1.7.549_TableS2.pdf]

Table S2. Specificity of enrichment of *in vitro* synthesized biotinylated *Arabidopsis thaliana* RNA on Streptavidin beads.

Transcript	UTP-RNA (Average Cp)	B16UTP-RNA (Average Cp)	Fold enrichment (x)
**ELF3 (779-926)**	33.39	29.4	15.9
**ELF3 (2631-2795)**	37.8	32.52	38.8

Average Cp values correspond to the average cross-point values from triplicate qPCR reactions
