# Supplementary material for: Genome-Wide Analysis of Nascent Transcription in Saccharomyces cerevisiae
Source: G3 (Bethesda). 2011 Dec 1;1(7):549–58. doi: 10.1534/g3.111.000810 (PMC3276176; doi:10.1534/g3.111.000810)
Supplement: Supporting Information [file supp_1.7.549_TableS3.pdf]

Table S3. Sequencing data acquisition and mapping statistics.

Library	Reads acquired	Reads mapped	Percent mapped	Unique, non-rRNA reads	Percent unique, non-rRNA reads
**NRO**	63,688,617	55,409,215	87.00%	2,492,414	4.50%
**RNA**	83,607,712	83,148,829	99.45%	939,582	1.13%
