# Supplementary material for: Genome-Wide Analysis of Nascent Transcription in Saccharomyces cerevisiae
Source: G3 (Bethesda). 2011 Dec 1;1(7):549–58. doi: 10.1534/g3.111.000810 (PMC3276176; doi:10.1534/g3.111.000810)
Supplement: Supporting Information [file supp_1.7.549_TableS4.pdf]

Table S4. GO term enrichment analysis for transcripts at top of the ranking by ratios of nascent transcription to transcript abundance (GOrilla).

	GO Term	Description	P-value	Enrichment	N	B	n	b
**Process**	GO:0015893	drug transport	5.11E-06	8.58	2383	13	171	8
	GO:0006414	translational elongation	7.02E-06	4.85	2383	172	40	14
	GO:0010033	response to organic substance	1.06E-05	1.83	2383	50	991	38
	GO:0006855	drug transmembrane transport	1.25E-05	17.94	2383	8	83	5
	GO:0000296	spermine transport	5.19E-05	238.3	2383	4	5	2
	GO:0015849	organic acid transport	7.35E-05	2.55	2383	44	468	22
	GO:0007165	signal transduction	1.00E-04	1.76	2383	117	578	50
	GO:0030447	filamentous growth	1.51E-04	1.77	2383	50	940	35
	GO:0006468	protein amino acid phosphorylation	1.55E-04	1.69	2383	64	923	42
	GO:0006075	1,3-beta-glucan biosynthetic process	1.64E-04	76.87	2383	2	31	2
	GO:0006074	1,3-beta-glucan metabolic process	1.64E-04	76.87	2383	2	31	2
	GO:0051274	beta-glucan biosynthetic process	1.70E-04	38.44	2383	6	31	3
	GO:0016310	phosphorylation	1.71E-04	1.65	2383	72	923	46
	GO:0051273	beta-glucan metabolic process	3.35E-04	32.94	2383	7	31	3
	GO:0019932	second-messenger-mediated signaling	3.58E-04	4.35	2383	15	329	9
	GO:0016049	cell growth	4.10E-04	1.85	2383	37	940	27
	GO:0015837	amine transport	4.31E-04	2.72	2383	30	468	16
	GO:0055085	transmembrane transport	4.56E-04	1.55	2383	182	575	68
	GO:0019236	response to pheromone	5.31E-04	2.16	2383	21	892	17
	GO:0065009	regulation of molecular function	6.13E-04	2.51	2383	77	259	21
	GO:0023052	signaling	6.69E-04	1.44	2383	137	909	75
	GO:0023033	signaling pathway	6.69E-04	1.44	2383	137	909	75
	GO:0015846	polyamine transport	7.73E-04	105.91	2383	9	5	2
	GO:0070783	growth of unicellular organism as a thread of	8.37E-04	1.8	2383	38	940	27
	GO:0044182	filamentous growth of a population of unicellular	8.37E-04	1.8	2383	38	940	27
	GO:0030811	regulation of nucleotide catabolic process	9.57E-04	5.88	2383	21	135	7
	GO:0033121	regulation of purine nucleotide catabolic process	9.57E-04	5.88	2383	21	135	7
	GO:0007187	G-protein signaling, coupled to cyclic nucleotide	9.63E-04	7.24	2383	4	329	4
	GO:0007188	G-protein signaling, coupled to cAMP nucleotide	9.63E-04	7.24	2383	4	329	4
**Function**	GO:0004871	signal transducer activity	2.85E-07	2.76	2383	35	617	25
	GO:0060089	molecular transducer activity	2.85E-07	2.76	2383	35	617	25
	GO:0005215	transporter activity	8.30E-06	1.65	2383	180	611	76
	GO:0004672	protein kinase activity	2.44E-05	1.78	2383	61	923	42
	GO:0003700	sequence-specific DNA binding transcription	2.82E-05	1.71	2383	64	979	45
	GO:0015297	antiporter activity	2.85E-05	74.47	2383	8	12	3
	GO:0000297	spermine transmembrane transporter activity	5.19E-05	238.3	2383	4	5	2
	GO:0022892	substrate-specific transporter activity	6.73E-05	1.65	2383	160	588	65
	GO:0004674	protein serine/threonine kinase activity	6.77E-05	1.77	2383	57	923	39
	GO:0022857	transmembrane transporter activity	7.08E-05	2.37	2383	153	197	30
	GO:0005099	Ras GTPase activator activity	1.80E-04	7.27	2383	17	135	7
	GO:0015238	drug transmembrane transporter activity	2.49E-04	11.96	2383	12	83	5
	GO:0005096	GTPase activator activity	2.85E-04	5.3	2383	30	135	9
	GO:0015203	polyamine transmembrane transporter activity	3.83E-04	136.17	2383	7	5	2
	GO:0016773	phosphotransferase activity, alcohol group as	4.51E-04	1.55	2383	88	923	53
	GO:0005088	Ras guanyl-nucleotide exchange factor activity	4.56E-04	4.56	2383	6	523	6
	GO:0043565	sequence-specific DNA binding	4.63E-04	1.6	2383	120	697	56
	GO:0015291	secondary active transmembrane transporter	5.99E-04	35.04	2383	17	12	3
	GO:0005097	Rab GTPase activator activity	7.62E-04	8.83	2383	10	135	5
	GO:0005275	amine transmembrane transporter activity	8.54E-04	2.53	2383	24	588	15
**Comp.**	GO:0005886	plasma membrane	9.90E-07	3.95	2383	175	69	20
	GO:0031224	intrinsic to membrane	3.57E-04	2.09	2383	460	72	29

N: Total number of genes. B: Total number of genes with specific GO term (background). n: Total number of genes in "target" set. b: Total number of genes with specific GO term in "target" set.
