# Supplementary material for: Genome-Wide Analysis of Nascent Transcription in Saccharomyces cerevisiae
Source: G3 (Bethesda). 2011 Dec 1;1(7):549–58. doi: 10.1534/g3.111.000810 (PMC3276176; doi:10.1534/g3.111.000810)
Supplement: Supporting Information [file supp_1.7.549_TableS5.pdf]

Table S5. GO term enrichment analysis for transcripts at bottom of the ranking by ratios of nascent transcription to transcript abundance (GOrilla).

	GO Term	Description	P-value	Enrichment	N	B	n	b
Process	GO:0006412	translation	4.45E-09	1.59	2383	177	922	109
	GO:0033044	regulation of chromosome organization	3.51E-04	38.23	2383	11	17	3
	GO:0006334	nucleosome assembly	3.59E-04	5.02	2383	14	271	8
	GO:0030004	cellular monovalent inorganic cation homeostasis	7.81E-04	4.79	2383	17	234	8
Func.	GO:0003735	structural constituent of ribosome	6.38E-08	2.41	2383	119	366	44
	GO:0005198	structural molecule activity	9.75E-06	1.89	2383	189	366	55
Component	GO:0044444	cytoplasmic part	3.51E-07	1.14	2383	1190	984	559
	GO:0044445	cytosolic part	4.82E-07	1.61	2383	136	905	83
	GO:0033279	ribosomal subunit	7.64E-07	1.85	2383	126	623	61
	GO:0032991	macromolecular complex	1.53E-06	1.45	2383	910	219	121
	GO:0043228	non-membrane-bounded organelle	8.46E-06	1.29	2383	417	897	202
	GO:0043232	intracellular non-membrane-bounded organelle	8.46E-06	1.29	2383	417	897	202
	GO:0044422	organelle part	3.19E-05	1.16	2383	1177	641	368
	GO:0044446	intracellular organelle part	3.19E-05	1.16	2383	1177	641	368
	GO:0015935	small ribosomal subunit	3.79E-05	3.14	2383	54	267	19
	GO:0022627	cytosolic small ribosomal subunit	4.48E-05	3.48	2383	41	267	16
	GO:0044424	intracellular part	6.52E-05	1.05	2383	2149	688	649
	GO:0005840	ribosome	7.19E-05	1.45	2383	175	875	93
	GO:0030529	ribonucleoprotein complex	9.06E-05	1.31	2383	309	898	152
	GO:0022625	cytosolic large ribosomal subunit	3.13E-04	1.74	2383	55	895	36
	GO:0005737	cytoplasm	8.73E-04	1.12	2383	1034	884	430

N: Total number of genes. B: Total number of genes with specific GO term (background). n: Total number of genes in "target" set. b: Total number of genes with specific GO term in "target" set.
