# Supplementary material for: Genome-Wide Analysis of Nascent Transcription in Saccharomyces cerevisiae
Source: G3 (Bethesda). 2011 Dec 1;1(7):549–58. doi: 10.1534/g3.111.000810 (PMC3276176; doi:10.1534/g3.111.000810)
Supplement: Supporting Information [file supp_1.7.549_TableS6.pdf]

Table S6. Sequencing data acquisition and mapping statistics of heatshock libraries.

Library	Reads acquired	Reads mapped	Percent mapped	Unique, non-rRNA reads	Percent unique, non-rRNA reads
**NRO (HS)**	19,918,770	11,423,271	57.35%	378,273	3.31%
**Total RNA (HS)**	70,159,160	69,502,678	99.07%	656,630	0.94%
