# Supplementary material for: Genome-Wide Analysis of Nascent Transcription in Saccharomyces cerevisiae
Source: G3 (Bethesda). 2011 Dec 1;1(7):549–58. doi: 10.1534/g3.111.000810 (PMC3276176; doi:10.1534/g3.111.000810)
Supplement: Supporting Information [file supp_1.7.549_FigureS9.pdf]

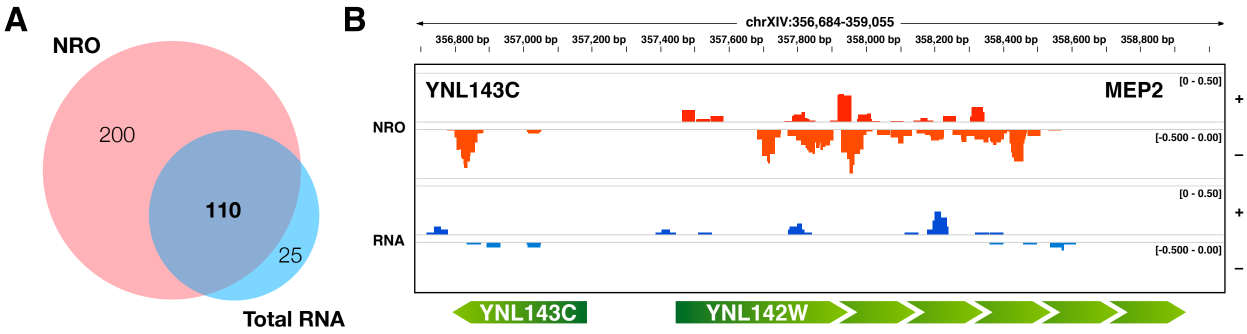

Figure S9. Antisense transcription in NRO and total RNA samples. (A) Venn diagram showing number of transcript models with significant antisense transcription in NRO (red) and total RNA (blue) samples and their overlap. As for expression analysis, the significance of antisense transcription within these models was calculated against the Poisson distribution of read depth per base expected from the background RNA-seq read depth in intergenic regions. A *P*-value significance cutoff of 0.01 was applied. **(B)** Prevalence of antisense transcription in NRO sample is exemplified by read depth in the *MEP2* gene.
